# The proteomic landscape of synaptic diversity across brain regions and cell types

**DOI:** 10.1016/j.cell.2023.09.028

**Published:** 2023-11-22

**Authors:** Marc van Oostrum, Thomas M. Blok, Stefano L. Giandomenico, Susanne tom Dieck, Georgi Tushev, Nicole Fürst, Julian D. Langer, Erin M. Schuman

**Affiliations:** 1 Max Planck Institute for Brain Research, Frankfurt am Main, Germany; 2 Max Planck Institute of Biophysics, Frankfurt am Main, Germany

**Keywords:** synapse, proteomics, synaptic proteins, synapse diversity, excitatory synapses, inhibitory synapses, dopaminergic synapses, fluorescence-activated synaptosome sorting, synaptic proteomics

## Abstract

Neurons build synaptic contacts using different protein combinations that define the specificity, function, and plasticity potential of synapses; however, the diversity of synaptic proteomes remains largely unexplored. We prepared synaptosomes from 7 different transgenic mouse lines with fluorescently labeled presynaptic terminals. Combining microdissection of 5 different brain regions with fluorescent-activated synaptosome sorting (FASS), we isolated and analyzed the proteomes of 18 different synapse types. We discovered ∼1,800 unique synapse-type-enriched proteins and allocated thousands of proteins to different types of synapses (https://syndive.org/). We identify shared synaptic protein modules and highlight the proteomic hotspots for synapse specialization. We reveal unique and common features of the striatal dopaminergic proteome and discover the proteome signatures that relate to the functional properties of different interneuron classes. This study provides a molecular systems-biology analysis of synapses and a framework to integrate proteomic information for synapse subtypes of interest with cellular or circuit-level experiments.

## Highlights

• Unveiled synaptic diversity: 1,800+ proteins define 18 distinct neuronal synapse types
• Correlation network analysis reveals excitatory and inhibitory protein communities
• Identified synaptic protein modules and proteomic hotspots for specialization
• Deep proteomic signatures of dopaminergic and cortical inhibitory subtype synapses

## Graphical abstract

**Figure:**
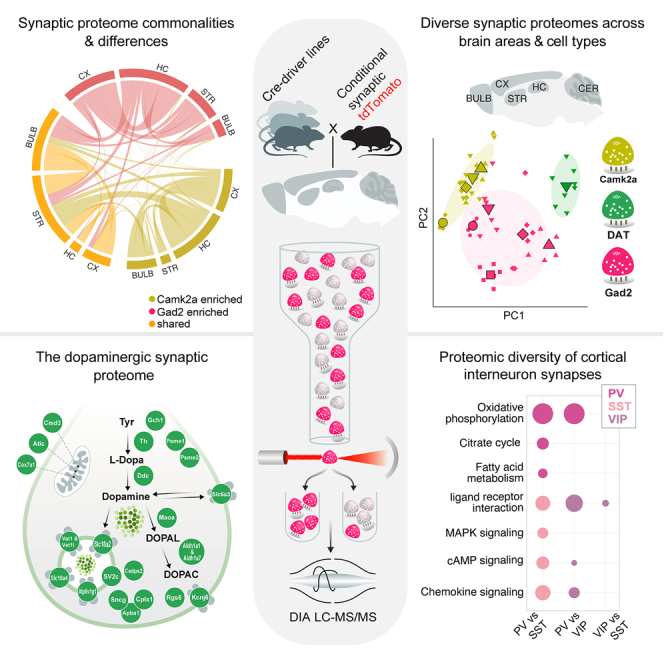

## Teaser

Systematic analysis unveils the diversity of synaptic proteomes and their organization, showcasing ∼1,800 unique synapse-enriched proteins, common synaptic modules, and specialized proteomic hotspots across 18 distinct synapse types.

## Introduction

The compartmentalization of biological processes in space and time within the single cell enables parallel processing of many reactions. Neurons, highly polarized cells, represent an extreme example of parallel processing—at their synapses, they compartmentalize information processing to communicate with thousands of other neurons.^1^ Synapses are plastic in structure and function, depending on their developmental and experiential history. This plasticity provides a molecular basis for learning and memory formation.^2^

The identity, copy number, post-translational modifications, and interactions of individual proteins largely determine the physiological properties of a given synapse.^3^ A growing body of evidence has found substantial structural and functional diversity of synapses.^4^ The complement of proteins that comprise average synaptic and sub-synaptic structures, such as the postsynaptic density (PSD) or synaptic vesicles, have been studied, but the proteome diversity that underlies different synapse types and states remains largely unexplored.^5^^,^^6^

The traditional biochemical isolation of synaptic terminals or synaptic elements^7^^,^^8^^,^^9^^,^^10^^,^^11^ yields synapse-enriched fractions (synaptosomes) that can be analyzed by mass spectrometry (MS)-based proteomics.^12^^,^^13^ These preparations, however, are often of low purity and contain a heterogeneous mixture of many synapse types along with contaminants. Immunoisolation and proximity-labeling strategies have been used to identify proteins in selected synaptic sub-compartments of some synapse types.^14^^,^^15^^,^^16^^,^^17^ In order to isolate a defined synapse population containing all synaptic elements from *in vivo* brain structures, Biesemann et al. introduced fluorescence-activated synaptosome sorting (FASS).^18^^,^^19^^,^^20^^,^^21^^,^^22^ This strategy utilizes fluorescent labeling of a synaptic protein to sort synaptosomes with high purity using a fluorescence-activated cell sorter and has been used to identify proteins that are enriched at growth cones^23^ or selected synapse types.^19^^,^^22^^,^^24^

Here, we used Cre-inducible knockin mice and FASS coupled to MS to investigate the diversity of the synaptic proteome across genetically defined synapse types and brain areas. We identified >1,800 unique synapse-enriched proteins that give rise to 18 different synapse-type-specific proteomes. This resource (https://syndive.org) reveals commonly shared synaptic protein modules as well as proteins that customize the proteomes of synapse populations related to their function, for example, at dopaminergic synapses in the striatum (STR) and subtypes of cortical interneurons.

## Results

**Figure 1:**
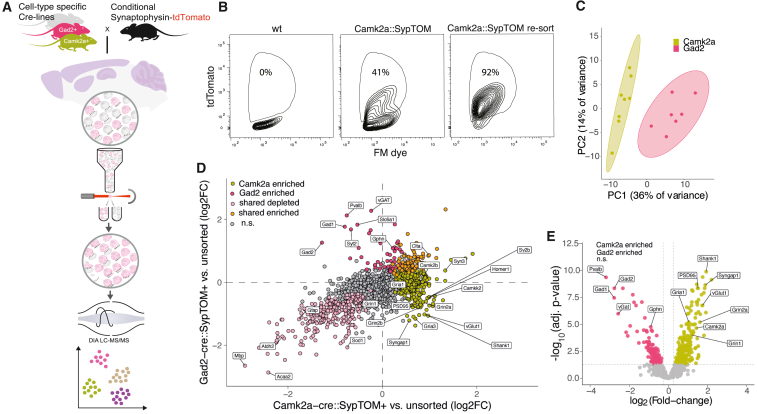
Synaptic diversity, proteomic discovery pipeline, and proof-of-principle (A) Crosses of different cell-type-specific Cre-driver lines (Camk2a+, Gad2+, Syn1+, Dat+, PV+, SST+, and VIP+) and a floxed synaptophysin-tdTomato line result in the cell-type-specific labeling of presynaptic terminals. Different indicated brain regions were microdissected, and synaptosomes were generated from each region. Fluorescence-activated synaptosome sorting (FASS) was used to purify the fluorescent, cell-type-specific population of synaptosomes. Then, each purified synaptosome population was subjected to data-independent acquisition (DIA) mass spectrometry, and the proteomes determined by statistical analysis of quantitative enrichment. (B) Gating strategy and sorting efficiency. FASS contour plots showing the relative density of the targeted tdTomato+ synaptic population in cortical synaptosomes prepared from wild-type mice (0%; left) or Camk2a::SypTOM mice (41%; middle). x and y axes represent fluorescence from a membrane dye (FM4-64) and tdTomato, respectively. Following the initial sorting run (middle), re-loading of the sorted synaptosomes indicated a high enrichment and purity (92%) of the Camk2a::SypTOM sample (right). (C) PCA showing the clear separation of Camk2a+ vs. Gad2+-sorted synaptosome proteomes. Individual data points represent replicates of the indicated groups. (D) Scatter plot comparing the differential enrichment of proteins in the Camk2a+-sorted and Gad2+-sorted synaptosomes to their control synaptosome precursor populations. Indicated are proteins that were significantly enriched in Camk2a+-sorted (lime green) or Gad2+-sorted synaptosomes (rose), significant in both populations (orange), or significantly de-enriched in both (pale pink). (E) Volcano plot comparing the proteins significantly enriched in Camk2a+-sorted (lime green) vs. Gad2+-sorted synaptosomes (rose). Some canonical marker proteins for excitatory and inhibitory synapses are highlighted. The y axis shows −log_10_ Benjamini-Hochberg adjusted p values. See also Figure S1.

**Figure S1:**
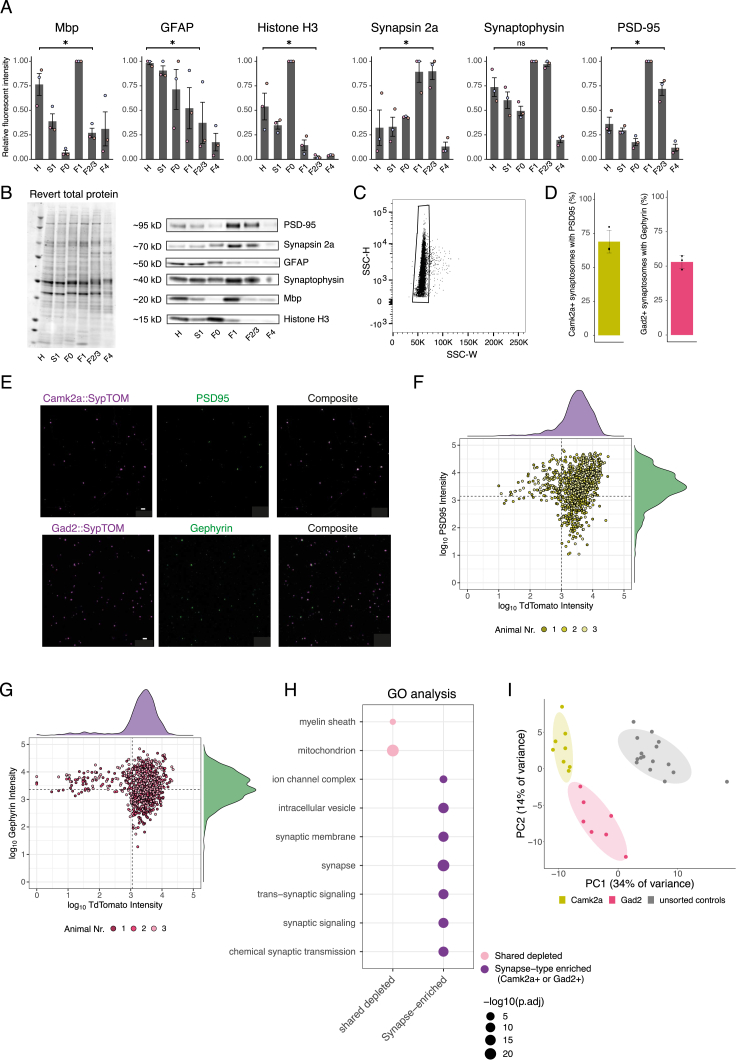
Characterization of the workflow to quantify synapse subtype-specific proteomes, related to Figure 1 (A) Contaminant proteins were significantly depleted in the (F2/3) synaptosomal fraction. Bar plots: relative fluorescent intensity across the different fractions of myelin basic protein 1 (paired one-tailed t test homogenate vs. F2/3, p = 0.023; n = 3), the glial marker glial fibrillary acidic protein (p = 0.048; n = 3), and the nuclear marker histone H3 (p = 0.032; n = 3). Synaptic proteins were enriched in the (F2/3) synaptosomal fraction; synapsin 2a (paired one-tailed t test homogenate vs. F2/3, p = 0.024; n = 3), synaptophysin (paired one-tailed t test homogenate vs. F2/3, p = 0.088; n = 3), and postsynaptic density protein 95 (paired one-tailed t test homogenate vs. F2/3, p = 0.017; n = 3). Error bars represent SEM. Relative fluorescent intensity is normalized for protein loading, local fluorescent background, and the maximum intensity of each sample. (B) Left: representative revert 700 total protein stain used to normalize for differences in protein loading. Right: representative immunoblot bands of the data shown in (A). (C) The first sorting gate (P1) used to exclude doublet particles based on side scatter. The particles in P1 were then gated according to Figure 1B to select for TdTomato+ particles. (D) Quantification of data shown in (E) for Camk2a+ synaptosomes and PSD95. Bar plots showing the percentage of TdTomato+ synaptosomes from Camk2a-cre crosses that contain a postsynaptic element (PSD95) and the same for inhibitory synaptosomes (Gad2-cre) using gephyrins as postsynaptic marker protein. n = 3 animals. (E) Representative images of spotted synaptosomes and their TdTomato fluorescence and PSD95 or gephyrin immunoreactivity for the data shown in (D). The composite contains both signals. Scale bars, 5 μm. (F) Scatterplot showing the identified particles of sorted Camk2a+ synaptosomes and their fluorescence for TdTomato and PSD95. For the estimation of particles that contained a postsynaptic element, shown in (D), the particles in the two right quadrants represent all TdTomato+ particles and form the denominator, and the particles in the upper right quadrant represent particles that are both TdTomato+ and PSD95+ and form the numerator. (G) Same as in (G), but for Gad2+ synaptosomes and the postsynaptic marker gephyrin. (H) Gene ontology (GO) analysis of the shared-enriched proteins and the union of shared, Camk2a-enriched and Gad2-enriched proteins (synapse-enriched) of Figure 1D. The selected GO terms highlight the enrichment of synaptic terms and de-enrichment of mitochondria and myelin. (I) PCA of Gad2+-enriched, Camk2a+-enriched, and unsorted control samples from the experiment displayed in Figure 1.

We developed a streamlined workflow to quantify the proteomes of synapses formed by different cell types in different brain areas. We used various Cre-inducible knockin mice expressing a fluorescently labeled presynaptic protein, synaptophysin-tdTomato (SypTOM), to target synapses that arise from cell types that use different neurotransmitters. We prepared and purified synaptosomes using FASS and coupled the output to a mass spectrometer to reproducibly quantify thousands of proteins (Figure 1A). We first tested the pipeline by comparing well-characterized Gad2-cre (cortical inhibitory) and Camk2a-cre (cortical excitatory) Cre-driver lines^25^^,^^26^ crossed with SypTOM mice (Figure 1A). We prepared synaptosomes from the cerebral cortex (CX)^27^^,^^28^ and verified that synaptosome fractions were enriched in pre- and postsynaptic proteins and depleted for non-synaptic contaminants (Figures S1A and S1B). We optimized the FASS^18^^,^^19^^,^^21^^,^^22^ strategy to define a population (P3) with a high tdTomato signal in combination with high membrane content, visualized by an FM dye (Figures 1B and S1C). Although control synaptosomes prepared from wild-type mice showed no detectable particles in the P3 gate, Camk2a::SypTOM synaptosomes constituted approximately 40% of the total particles (Figure 1B). Re-analysis of sorted synaptosomes by flow cytometry routinely led to a purity of ∼80%–95% (Figure 1B). Immunolabeling of individually spotted sorted synaptosomes revealed that most synaptosomes contained a postsynaptic element, and ∼65% of Camk2a::SypTOM synaptosomes were labeled for the excitatory postsynaptic marker PSD95, whereas ∼50% of the Gad2::SypTOM synaptosomes were labeled for inhibitory postsynaptic marker gephyrin (Figures S1D–S1G). In this proof-of-principle experiment, we sorted ∼20 million cortical synaptosomes from 8 Camk2a::SypTOM and 8 Gad2::SypTOM mice and quantified their proteomes.^29^^,^^30^ For each mouse, we additionally processed control samples consisting of the same number of particles from the precursor synaptosome population (the input to sorting). To achieve this, we purified all particles with a membrane dye signal intensity above the noise threshold but independent of tdTomato signal, resulting in an unsorted control sample. Overall, we quantified >2,300 protein groups. We defined the individual synaptic proteomes as the complement of proteins that were significantly enriched relative to their respective control fractions (Tables S1A and S1B). We found that the proteomes of Camk2a::SypTOM and Gad2::SypTOM synaptosomes were enriched in synaptic proteins and clearly separated from unsorted synaptosomes in a principal-component analysis (PCA) (Figures 1C, 1D, S1H, and S1I). We examined the proteins with the highest enrichment in the direct quantitative comparison of Camk2a::SypTOM and Gad2::SypTOM proteomes and observed that they represent almost exclusively established marker proteins for cortical excitatory and inhibitory synapses (Figures 1D and 1E; Table S1C). We compared the 20 most enriched proteins (top ∼10%) for Camk2a::SypTOM with the SynGO synaptic protein database^5^ and found 19 with prior synaptic annotation. These data demonstrate that this workflow can identify synaptic proteomes from a small number of purified synapses.

**Figure 2:**
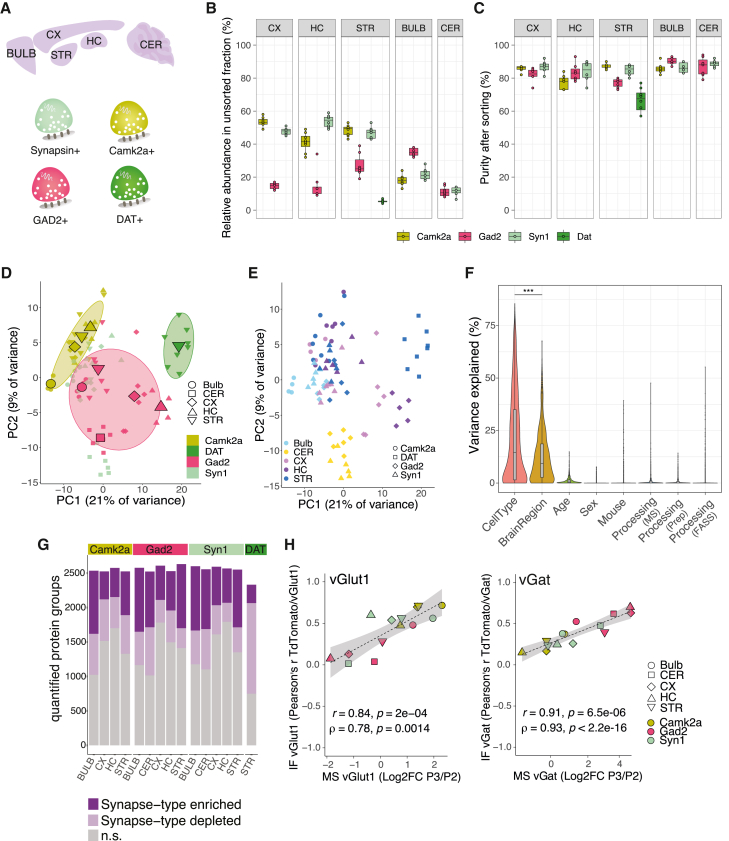
Synaptic proteomic diversity across brain areas and cell types (A) Scheme indicating the brain areas that were microdissected from Camk2a::SypTOM, Gad2::SypTOM, Syn1::SypTOM, or Dat::SypTOM mice and then introduced to the pipeline. (B) Plot indicating the relative abundance of each fluorescently labeled synaptosome type in the crude synaptosome fraction generated from the brain areas indicated (x axis). (C) Plot indicating the purity of each fluorescently labeled synaptosome type from the brain areas indicated (x axis) after FASS. (D) Principal-component analysis (PCA) in which the cell type clusters are highlighted. Small symbols denote individual biological replicates, and large symbols denote averages of each synapse subtype. (E) PCA in which the brain regions are highlighted. Symbols denote individual biological replicates. (F) Violin plots depicting the percentage of the variance explained by individual covariates. ^∗∗∗^p < 0.001; t test, n = 1,022. (G) Number of protein groups quantified for each synapse subtype, grouped by cell types and brain regions. Shown are significantly enriched and de-enriched groups (see STAR Methods), as well as protein groups that are not significantly different between the groups. (H) Correlation between immunofluorescence and mass spectrometric measurements for vGat and VGlut1 proteins across the 15 synapse types. x axis shows mass spectrometric measurements for vGat and VGlut1 protein. y axis indicates immunofluorescence measurements for vGat and VGlut1 proteins. The immunofluorescence data are described in Figure S2. See also Figures S2 and S3.

**Figure S2:**
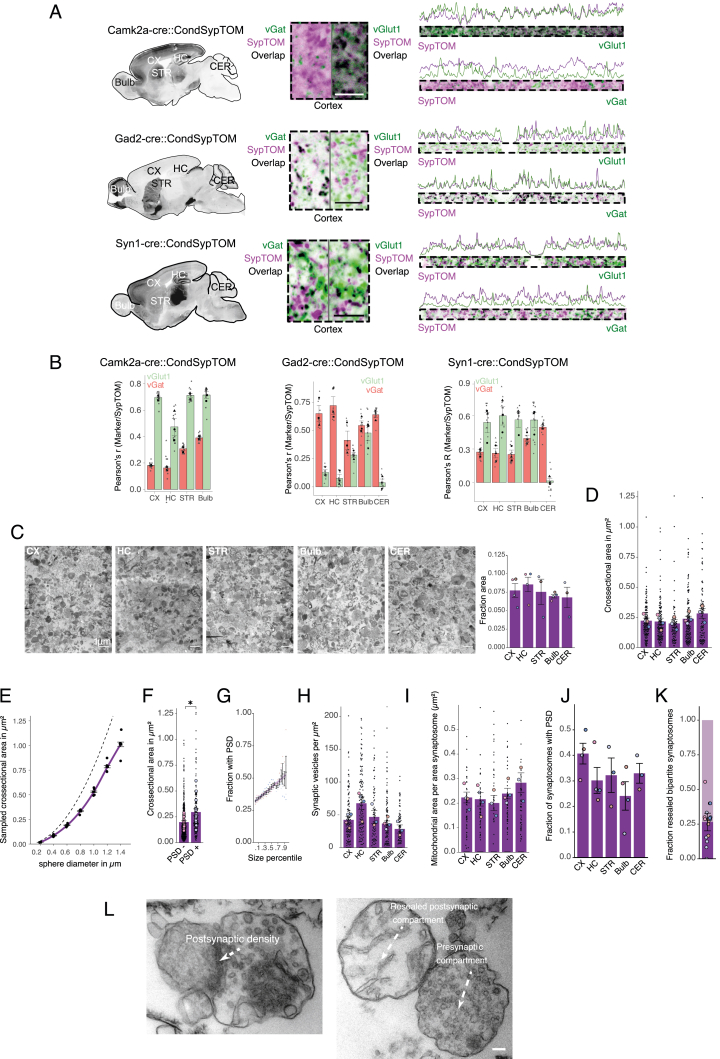
Assessment of synaptic populations that are labeled by Camk2a-cre, Gad2-cre, and Syn-cre crosses, related to Figure 2 (A) Left: sagittal overview of SypTOM expression in a Camk2a::SypTOM, Gad2::SypTOM, and Syn1::SypTOM. Middle: magnified representative image of an immunofluorescent co-staining for the inhibitory synapse marker solute carrier family 32 member 1 (vGat) and the excitatory synapse marker solute carrier family 17 member 7 (vGlut1), both in green, and of SypTOM expression, in purple, for the three mouse lines. Overlap is depicted in black. Right: fluorescence intensity analysis showing the relative spatial coincidence of the SypTOM with vGlut1 and vGat for the three mouse lines. Scale bars, 5 μm. (B) Pearson’s correlation between SypTOM fluorescence and the vGat (red) and vGlut1 (green) show distinct labeling in the CX and HC, and to a lesser extent in STR and Bulb for Camk2a::SypTOM. The Gad2::SypTOM mouse shows relatively high labeling for inhibitory synapses in the cortex, hippocampus, and cerebellum and lower labeling in the striatum and olfactory bulb. The Syn1-cre mouse shows overlapping synaptic labeling with both vGat and vGlut1 in all brain regions except for the cerebellum, where predominantly inhibitory synapses are labeled. Large symbols, average per mouse; small symbols, individual images. Error bars signify the standard error of the mean (SEM). n = 3 animals, 4 images per brain region for every marker and mouse. (C) Synaptosome density in electron micrographs was comparable across the analyzed brain regions. Displayed are representative EM tile scans (acquired with a 31,500× magnification) of representative synaptosome fractions originating from the cortex (CX), hippocampus (HC), striatum (STR), olfactory bulb (Bulb), and cerebellum (CER). Scale bars, 1 μm. Synaptosomes were identified as round structures containing synaptic vesicles. The bar chart shows the fractional area of the image, which was annotated as synaptosome. We found no significant differences in synaptosome abundance across the analyzed brain regions (repeated measures ANOVA, p = 0.95; CX: n = 4, HC: n = 4; STR: n = 3; Bulb: n = 4; CER: n = 4; n = number of animals). (D) Synaptosomes from different brain areas did not exhibit significantly different sizes. Cross-sectional area (μm^2^) of synaptosomes originating indicated brain areas were not significantly different (repeated measures ANOVA, p = 0.21; n (number of animals) = 4, 4, 3, 4, and 4 for CX, HC, STR, Bulb, and CER, respectively). Large symbols, average cross-sectional area from 30 synaptosomes; small symbols, individual synaptosomes. (E) Synaptosome cross-sectional sampling results in an underrepresentation of the true size. Therefore, we estimated the extent of underrepresentation of the true synaptosomes size assuming a spherical shape (see STAR Methods). We randomly sampled 30 planes from a sphere with the indicated diameter (n = 4) and plotted the obtained average cross-sectional area (black dots). Connecting the sampled data points provides the purple line, which was used to estimate the synaptosome diameter from the experimentally sampled mean cross-sectional area. The dashed line indicates the maximal cross-sectional area per diameter, assuming sphericity without cross-sectional sampling. (F) Synaptosomes that possessed a postsynaptic density (PSD) have a higher cross-sectional area, indicating that their cross-sectional sampling was closer to the maximum (paired one-tailed t test synaptosomes with vs. without PSD, p = 0.031; n = 4; brain regions pooled). (G) The fraction of synaptosomes with a PSD attached scales with cross-sectional synaptosome area percentile; plotted is the fraction of synaptosomes with a PSD for different percentiles. Line indicates 99% confidence interval. (H–J) (H) The number of synaptic vesicles, mitochondrial area (I), or fraction with a visible PSD (J) did not differ significantly between synaptosomes across the analyzed brain regions (normalized for synaptosome size, repeated measures ANOVAs; I: p = 0.083, J: p = 0.337, K: p = 0.15, same samples as in A). (K) Bipartite synaptosomes (synaptosomes containing both a pre- and postsynaptic element) have a resealed postsynaptic compartment in ∼25% of the cases, and the colors of the data points indicate that the synaptosomes originate from the same mice, albeit different brain regions. All error bars indicate SEM. (L) Representative images of synaptosomes after sorting acquired at a magnification of 4,100×. Recognizable ultrastructural features are annotated. Scale bars, 100 nm.

We applied the above pipeline to investigate the proteomic diversity of 15 different major synapse subtypes using four Cre-driver lines representing different cell types and microdissection of five different brain areas (CX, hippocampus [HC], STR, olfactory bulb [Bulb], and cerebellum [CER]) (Figure 2A). Besides Camk2a- and Gad2-cre, we included Syn1-cre (synapsin 1), a line that is independent of neurotransmitter type, and Dat-cre, representing the synapses that use the modulatory neurotransmitter dopamine. In control experiments, we first comprehensively assessed the degree to which a given Cre-line labeled the expected synaptic population in a given brain area (Figures S2A and S2B). We found that in the CX and HC, Camk2a::SypTOM and Gad2::SypTOM mice showed specificity for excitatory and inhibitory synapses, respectively. In the STR and Bulb, however, Camk2a::SypTOM and Gad2::SypTOM labeled both distinct and overlapping synaptic populations (Figures S2A and S2B). Finally, two-dimensional (2D) electron microscopy (EM) showed that synaptosome preparations from different brain regions did not differ in their synaptosome abundance, in the size or presence of synaptic vesicles, or in synaptic mitochondria or postsynaptic structures between brain regions (Figures S2C–S2L).

**Figure S3:**
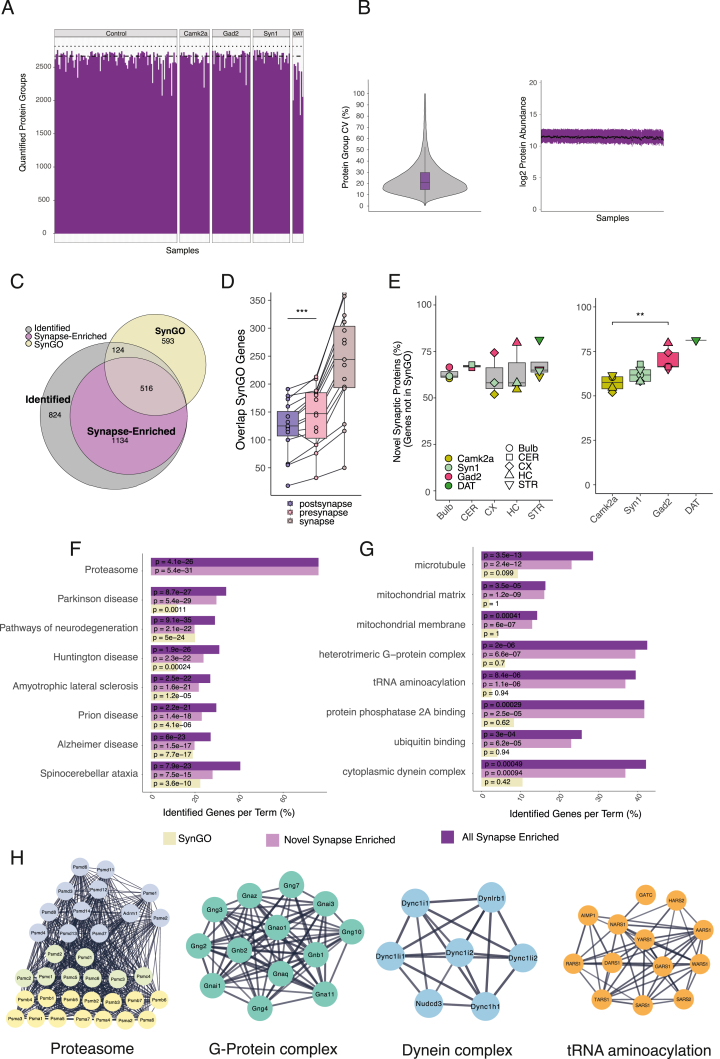
Comparison of synapse-enriched proteins with the SynGO database, related to Figure 2 (A) Bar plot showing the number of quantified protein groups per sample, sorted by cell types. Dash-dotted line indicates the mean number of quantified protein groups, and dotted line indicates the total number of quantified proteins in the experiment. 23% of the quantified protein groups contained a membrane domain; in UniProtKB/Swiss-Prot (*Mus musculus*), 26% of proteins are annotated with a membrane domain. (B) Left: log_2_ protein intensities across all mass spectrometry (MS) samples. Black dots indicate median intensity; upper and lower hinges the 25th and 75th percentiles. Right: violin plot showing the distribution of protein group coefficient of variation (CV) within conditions (synapse types), and the median CV was ∼20%. Boxplot indicates the median, 25th and 75th percentiles. (C) Euler diagram showing overlap between all quantified proteins, proteins that were identified as synapse-enriched, and the SynGO database. 81% of the SynGO-annotated proteins that were identified in our experiments were quantified as synapse-enriched. (D) Boxplot showing overlap with SynGO-annotated genes for all synapse types and the terms “synapse,” “presynapse,” and “postsynapse.” Each dot represents a synapse subtype. Overlap with SynGO was significantly higher for the term presynapse compared with the term postsynapse. n = 15, paired t test, ^∗∗∗^p < 0.001. (E) Boxplots showing percentage of novel synaptic proteins distinguished by brain region (left) or by cell type (right). We detected significantly more novel synaptic proteins in Gad2+ synapses compared with Camk2a+ synapses. n = 4 (Camk2a), 5 (Gad2), t test, ^∗∗^p < 0.01. (F) Bar plot showing selected significantly enriched KEGG pathways for all genes in SynGO, all synapse-enriched proteins, and the proteins identified as synapse-enriched but not previously annotated in SynGO. x axis shows the percentage of genes that were identified of the total number of genes that are associated with each term. Text indicates p value of the respective group. (G) The same as in (D) but for selected gene ontology terms. (H) Visualization of selected proteins that are associated with enriched terms in the “novel synapse-enriched” group using data from the STRING interaction database. Edges represent stringdb score >0.7 (high confidence).

The fluorescently labeled synaptosome populations that we studied originally comprised ∼5% to ∼50% of a given brain region’s total crude synaptosome population (Figure 2B). We purified all synapse types (but one, Dat::SypTOM) to greater than 75% purity, on average (Figure 2C). For each synapse subtype, we obtained ∼10 Mio sorted (P3 gate) particles and the same number of matched control particles from at least 5 mice. We processed the sorted synaptosomes for label-free quantitative proteome analysis. Overall, we quantified >2,800 protein groups with a high reproducibility (median coefficient of variation [CV] ∼20%) among biological replicates (Figures S3A and S3B). In order to define the individual synaptic proteomes, we determined the complement of proteins that were quantitatively enriched in each synapse type (Table S2A), compared with its own precursor (unsorted) synaptosome population from the same brain region. In a PCA, the different synaptic proteomes were distinguished by both cell types (Figure 2D) and brain regions (Figure 2E). Which feature, cell type or brain region, exerts the greatest influence on the synaptic proteomes? Overall, the cell types explained more of the total variance than the brain regions (Figure 2F), and other factors like the age or sex of the animals had a negligible influence on the observed variance (Figure 2F). In total, we allocated >10,000 protein groups to the 15 synaptic proteomes (Figure 2G) and identified >1,800 unique protein groups that were enriched in at least one of the 15 synapse subtype proteomes (Figure 2G; Table S2A). We created an interactive web tool that allows one to query the abundance and localization of individual proteins in individual synapse types (The Synaptic Diversity Hub, https://syndive.org/). We compared the enrichment of the vesicular GABA transporter (vGat) and vesicular glutamate transporter 1 (VGlut1) proteins from MS with those obtained by immunofluorescence on brain slices (Figure S2B) and found high correlation coefficients of 0.84 and 0.91 for VGlut1 and vGat, respectively (Figure 2H), validating the specificity of these synaptic proteomes.

To what extent do the proteins identified here represent previously known synaptic molecules? To address this, we compared the union of all the unique synapse-type-enriched proteins with the synaptic protein database SynGO.^5^ Overall, there was very good agreement between the two datasets: ∼80% of the identified SynGO-annotated proteins were classified as synapse-enriched in our analysis (Figures S3C–S3E). Furthermore, we identified >1,000 new synapse-enriched protein groups (Table S2B). These novel synapse-enriched proteins are significantly associated with various disease-related pathways and underlie different cellular functions such as G-protein signaling, protein degradation, or tRNA aminoacylation (Figures S3F–S3H). When each synaptic proteome was analyzed individually, each one was significantly enriched in SynGO terms (Table S2C). As might be expected from the localization of the SypTOM protein, we found significantly more proteins annotated as presynaptic than postsynaptic across the 15 synapse types (Figure S3D). We also identified significantly more novel synaptic proteins at Gad2::SypTOM synapse types as compared to Camk2a::SypTOM (Figure S3E) and the most novel proteins at Dat::SypTOM synapses, presumably reflecting a bias of the published synaptic protein literature toward excitatory synapses.

**Figure S4:**
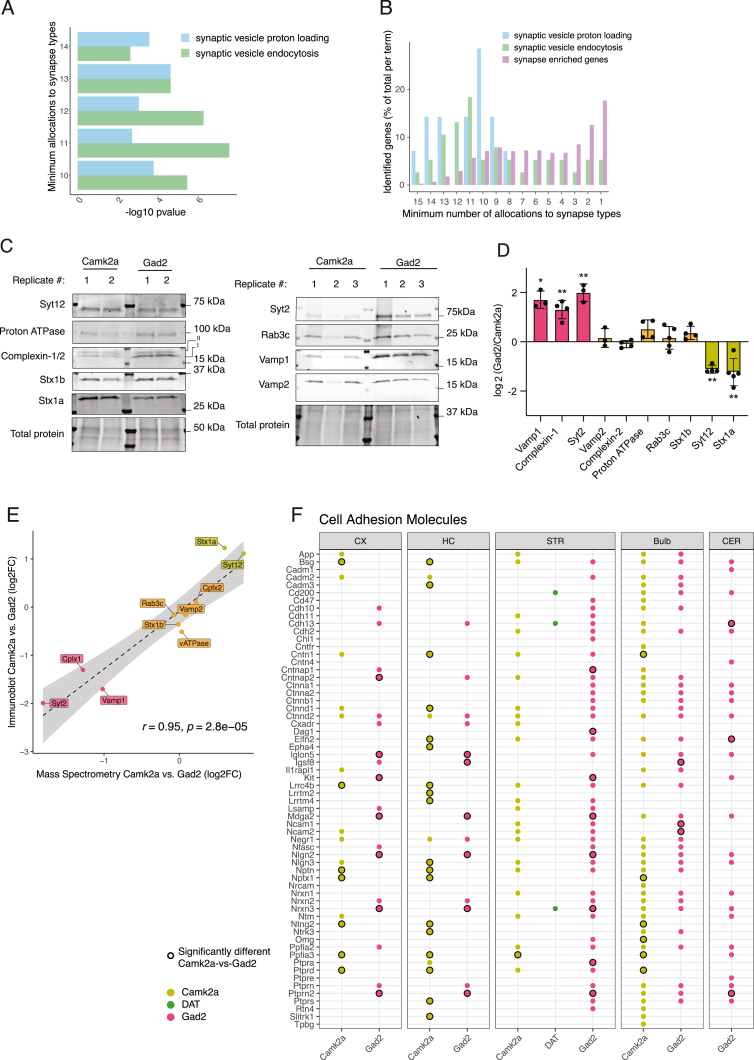
Shared and subtype-specific enrichment of synaptic proteins, related to Figure 3 (A) Bar plot showing SynGO enrichment p values for the proteins that were identified in a minimum of 10, 11, 12, 13, or 14 of the total of 15 synapse types. (B) Distribution of proteins that were associated with the SynGO terms synaptic vesicle proton loading or synaptic vesicle endocytosis depending on groups defined by the minimum number of synapse types with which they were associated. The distribution for all synapse-enriched proteins is plotted for comparison. (C) Immunoblot validation. Representative immunoblots of sorted Camk2a+ and Gad2+ synaptosomes. Each lane corresponds to 25 Mio sorted synaptosomes. Displayed are two independent biological replicates (animals) per synapse type. (D) Histogram of immunoblot quantifications. ^∗^p ≤ 0.05, ^∗∗^p ≤ 0.01, unpaired t test, two-tailed; the data reported are mean and SD. n = 3 for Vamp1, Vamp2, and Syt2; n = 4 for Syt12, Stx1b, complexin-1/2, and proton ATPase; n = 5 for Rab3c and Stx1a. (E) Correlation plot of immunoblot and mass spectrometry analysis. Dots represent log_2_FC measurements of either technology comparing Camk2a+ vs. Gad2+ for the indicated proteins. The dashed line represents a linear regression of the depicted data points. The gray area represents the standard error of the regression. Immunoblotting and mass spectrometry show a very high significant correlation (Pearson’s r = 0.95, p = 2.8e−5). (F) Heatmap for transsynaptic cell-adhesion molecules^32^ showing their variable presence in Camk2a+ and Gad2+ synaptic proteomes across brain regions. Black border around dots indicates a significant difference.

**Figure 3:**
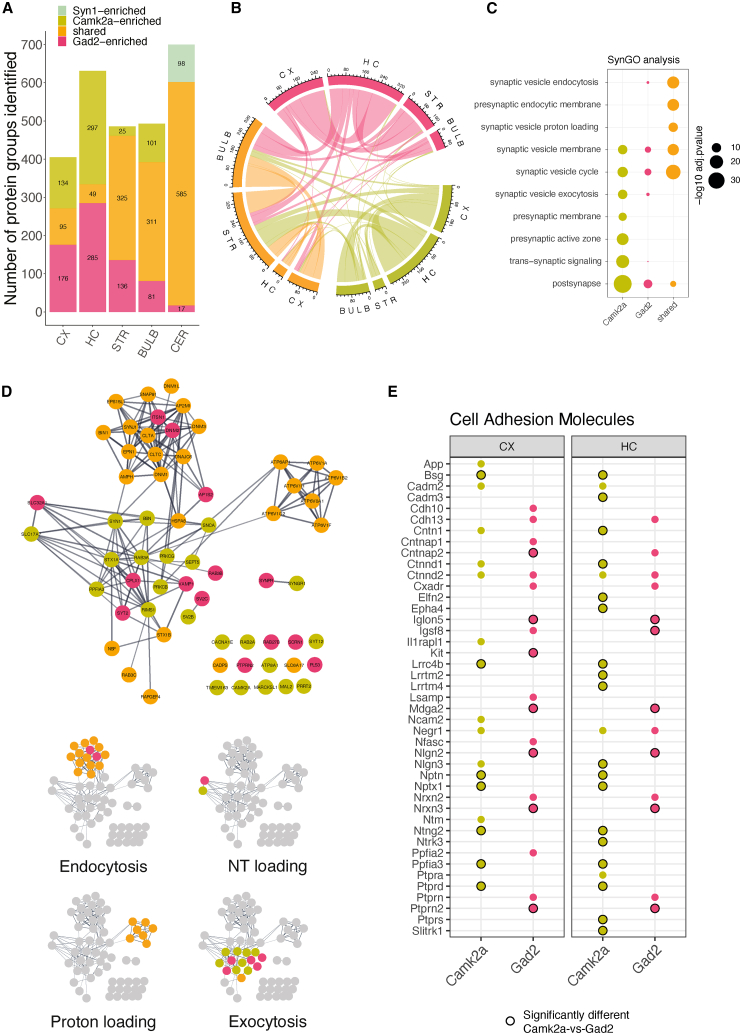
Synaptic proteome commonalities and differences (A) Bar plot showing proteins significantly enriched in the direct quantitative comparison of Camk2a+ vs. Gad2+ synaptic proteomes for each brain region. Shared proteins are defined as significantly enriched in both Camk2a+ and Gad2+ vs. control synaptosomes and not significantly different between Gad2+ and Camk2a+. Note the increased number of shared proteins in brain regions where Camk2a-cre and Gad2-cre label exclusive as well as overlapping synapse types (Figures S2A and S2B). The cerebellum lacked detectable tdTomato signal in the Camk2a::SypTOM mouse and therefore was replaced by the Syn1+ proteome. (B) Chord diagram of intersections between the groups defined in (A), with the 3 colors representing Camk2a+-enriched (green), Gad2+-enriched (red), or enriched in both (yellow). The arcs indicate overlapping proteins between the two connected groups. Note that there are few intersections (∼1% of synapse-enriched proteins) between Camk2a and Gad2 relative to Gad2 with shared and Camk2a with shared. (C) Dot plot of SynGO analysis results for shared and cell-type-specific enriched proteins. Depicted are selected significantly enriched SynGO terms of Camk2a-enriched, Gad2-enriched, and shared groups from cortex. (D) Protein interaction network of synaptic vesicle cycle proteins for cortical Camk2a, Gad2, or shared-enriched groups. Proteins with SynGO annotation for the synaptic vesicle cycle are displayed. Edges represent a stringdb score >0.7 (high confidence).^31^ Proteins that are associated with the significantly enriched terms “synaptic vesicle endocytosis,” “synaptic vesicle exocytosis,” and “synaptic vesicle proton loading” (data shown in C) are indicated on the left. (E) Heatmap for transsynaptic cell-adhesion molecules^32^ showing their presence in cortical and hippocampal Camk2a+ and Gad2+ synaptic proteomes. Black border color indicates significant enrichment in the direct comparison of Camk2a vs. Gad2 proteomes. Note that very few adhesion proteins were common in Camk2a+ and Gad2+ synaptic proteomes (<5%), and the majority show synapse-type-specific localization. See also Figure S4.

Are there protein modules that are shared among the different synapse types? To address this, we selected proteins that were detected in at least 14 of the 15 synapse types and performed an enrichment analysis using the SynGO database. We identified a significant enrichment of synaptic vesicle vesicular ATPases (vATPases) as well as proteins mediating synaptic vesicle endocytosis (Figure S4A). Both terms were also significantly enriched when selecting proteins that were detected in minimally 10, 11, 12, or 13 synapse-type proteomes (Figure S4B). The enriched proteins include, for example, clathrin (Clta, Cltb, and Cltc) and proteins of the associated adapter protein complex (AP-2) or the vATPase Atp6v1f, which were enriched at every synapse type. Next, we quantitatively compared Gad2::SypTOM with Camk2a::SypTOM synaptosomes and defined Gad2-enriched, Camk2a-enriched, and shared protein groups in each brain region (Figure 3A). This analysis revealed proteins that were quantitatively enriched at either synapse type over the other (Camk2a-enriched or Gad2-enriched) or enriched in both types over controls and not significantly different between the types (shared). In general, we observed very little overlap (∼1% of all synapse-enriched proteins) between Gad2::SypTOM-enriched and Camk2a::SypTOM-enriched proteins, indicating that across all brain regions, excitatory and inhibitory synapses have subsets of mutually exclusive synaptic proteins (Figure 3B; see further analysis below). However, since Gad2-cre and Camk2a-cre label different neuron populations in different brain regions (Figure S2B), there were shared proteins in some brain regions available for analysis (Figure 3B). To examine in more detail the shared proteins, we focused on the shared proteins of the CX (where Gad2::SypTOM and Camk2a::SypTOM label specifically excitatory and inhibitory synapses, respectively; Figures S2A and S2B). Within the shared proteins, we again observed an overrepresentation of synaptic vesicle endocytosis and synaptic vesicle proton loading, whereas exocytosis and presynaptic active zone proteins were absent or minimally represented in the shared fraction but overrepresented in the Gad2-enriched and/or the Camk2a-enriched fraction (Figures 3C and 3D).

We constructed a protein-protein interaction network^31^ using the proteins associated with the enriched terms and revealed that protein modules that represent different steps of the synaptic vesicle cycle were enriched in either shared or subtype-specific synaptic proteins (Figure 3D). We observed intriguing patterns among certain protein families, such as the Rab, Vamp, Stx, or Syt groups, where related family members displayed distinct type-specific enrichment at excitatory or inhibitory synapses. For a subset of these proteins, we conducted immunoblotting experiments that confirmed these data (Figures S4C–S4E), and notably, we observed an excellent correlation between immunoblot and MS analyses (Pearson’s r = 0.95). This diversity suggests that these proteins may differentially modulate the same synaptic molecular process, potentially influencing synapse-type-specific characteristics.

Cell-adhesion molecules (CAMs) are known to contribute to the specificity in synaptic connections.^3^ We retrieved a curated list of transsynaptic CAMs^32^ and asked whether these proteins show preferential enrichment at excitatory or inhibitory synapse types. Indeed, in the CX and HC, we found >95% of the CAMs were exclusively enriched at either excitatory or inhibitory synaptic proteomes (Figures 3E and S4F), indicating a highly cell-type-specific localization. Taken together, the above analyses highlight endocytosis and vATPases as generic synaptic protein modules that are used by synapses independent of neurotransmitter type, whereas the exocytosis machinery, the presynaptic active zone, transsynaptic adhesion molecules, and postsynaptic protein modules make use of different sets of proteins for different synapse types.

**Figure 4:**
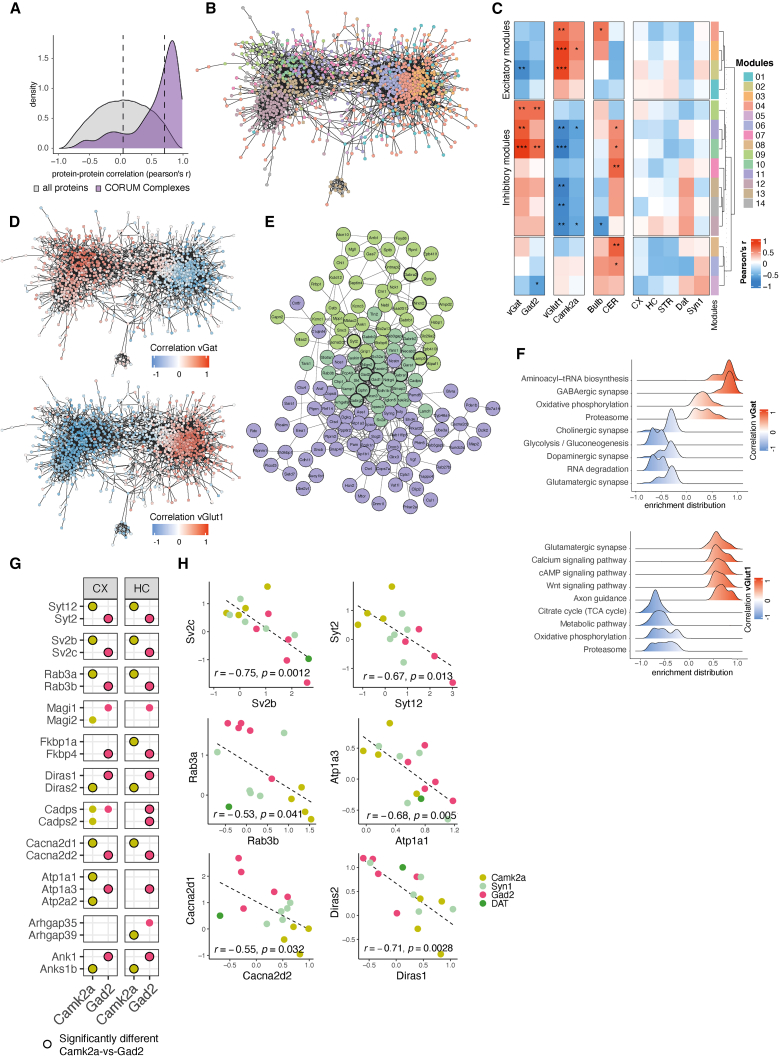
The synaptic protein-protein correlation network reveals protein communities (A) Density plot of pairwise protein-protein abundance profile correlations (Pearson’s r) for protein pairs that are annotated members of the same protein complex (purple)^34^ and for random protein pairs (gray) as control. Proteins of the same complex exhibited a highly co-regulated abundance profile across the 15 synapse types, whereas random protein pairs showed no correlation on average. Dashed lines denote median values for each group. The left tail of the distribution depicts negatively correlated protein pairs previously linked to a shared complex, which may arise because of differences in subcellular or cell-type-specific protein complexes or the formation of different complexes across various synapse types. (B) Community network of protein-protein correlations. The network represents a visualization of the adjacency matrix used for WGCNA. The nodes are synapse-enriched proteins, and they are connected by edges that represent the abundance profile correlation of the two nodes they connect. Specifically, edges represent adjacency based on biweight midcorrelation and are filtered for weights >0.3, meaning negative and low correlations are not considered for visualization of the network. Protein nodes are colored according to their associated protein module. Only synapse-type-enriched proteins without missing values are used for the analysis (1,557 proteins). (C) Heatmap of module correlations with synaptic traits. Protein module Eigen proteins were correlated with the following traits of the 15 synapse types: cell type, brain region, and immunofluorescence for vGat and VGlut1. Protein module Eigen proteins are protein abundance profiles that are representative for the proteins in their module (specifically, the first principle component of the module). ^∗^p < 0.05; ^∗∗^p < 0.01; ^∗∗∗^p < 0.001; Pearson correlation. (D) Network from (B) with the protein nodes colored for correlation with vGat immunoreactivity (top) and VGlut1 immunoreactivity (bottom), revealing the nature of the two protein communities. The proteins of the left community showed a correlation with the vGat and an anti-correlation with VGlut1, whereas the proteins of the right community showed a correlation with vGlut1 and an anti-correlation with vGat. (E) Subnetwork from (B) depicting only the proteins from modules that were significantly correlated with vGat immunofluorescence (modules 9, 10, and 11). Selected proteins of interest are highlighted with an increased border width. (F) Ridge plots for pathways enriched in VGlut1 and vGat protein communities showing enrichment distribution for core-enriched genes of selected significantly enriched terms. GSEA was conducted using the Kyoto Encyclopedia of Genes and Genomes (KEGG) pathway database^89^^,^^67^ and proteins ranked by their correlation with vGat and VGlut1 immunofluorescence. (G) Heatmap for selected negatively correlated protein pairs showing presence in cortical and hippocampal Camk2a+ and Gad2+ synaptic proteomes. Black border color indicates a significant enrichment in the direct comparison Camk2a vs. Gad2 proteomes. The full list of negatively correlated protein pairs is in Table S3 and displayed in Figure S5. (H) Correlation plots for selected negatively correlated protein pairs. Dots represent the log_2_fold-change (FC) for each protein on the x and y axes compared with unsorted controls for each synapse type. The color indicates the cell type underlying the synapse type. The dashed lines represent a linear regression through the indicated data points. Pearson’s r and statistical significance are indicated at the bottom of each plot. See also Figure S5.

Are there specific protein modules that are associated with synapses that use different neurotransmitters or are associated with different cell types or brain regions? To address this, we used a protein-protein weighted correlation network analysis (WGCNA)^33^ to identify protein modules that show correlated abundance patterns across the 15 synapse types. We first asked a simpler question: do proteins of the same complex or functional unit exhibit correlated expression levels? We found a significantly higher median correlation for proteins that are subunits of the same protein complex (Pearson’s r = 0.65)^34^ as compared with random protein pairs (Pearson’s r = 0.03) (Figure 4A). For example, the proteasome subunits Psma1 and Psma7 exhibited highly correlated abundance (r = 0.97); similarly, vGat and Gad2 (the essential synaptic GABA synthesis enzyme) were correlated with a near-perfect coefficient of 0.98. We next constructed a protein-protein correlation network using all synapse-enriched proteins and identified 14 protein modules using WGCNA (Figure 4B; Table S3). We discovered that the resulting protein network featured two main opposing clusters, defining two highly connected protein communities. To identify the nature of these protein communities, we correlated all protein modules with traits of the synapse subtypes, including immunofluorescence for vGat and VGlut1 (Figure S2B), cell types, and brain regions. Although most brain regions and the cell type Syn1 showed no correlation with any protein module, we identified three inhibitory protein modules that were significantly correlated with vGat and three excitatory protein modules that were significantly correlated with VGlut1 (Figure 4C). We found that both the excitatory and inhibitory modules were located at the center of each protein community and that each community was characterized by a high correlation or anti-correlation with vGat or VGlut1 (Figure 4D; Table S3). In summary, we constructed a synaptic protein-protein correlation network that revealed two distinct protein communities representing the proteomes associated with excitatory or inhibitory neurotransmitters across distinct cell types and brain regions.

Can we identify the key proteins for the synaptic proteomes of glutamatergic and GABAergic synaptic proteomes based on the network topology? We identified 188 and 315 proteins that were significantly correlated with vGat or VGlut1 expression, respectively, with correlation coefficients ranging from moderate (∼0.5) to very high (∼0.95) (Table S3). Proteins with high correlation coefficients were also among the most connected nodes within each community (Figure 4D). We inspected the network topology of the protein modules with a significant correlation for vGat and found that the core module (module 10) contained established inhibitory marker proteins, including Gad-1, Gad-2, Gaba transporter-1, Neuroligin-2, multiple GABA-A receptor subunits, and the inhibitory postsynaptic scaffolding protein gephyrin (Figure 4E). Notably, module 10 contained proteins from both the pre- and postsynaptic compartments, indicating a tight co-regulation of synaptic architecture across the synapse. Module 9, by contrast, was characterized by a moderate but significant correlation with vGat. In this module, we detected proteins that have been previously associated with subtypes of GABAergic neurons or synapses, for example, Syt-2,^35^ Lamp-5, cannabinoid receptor 1 (Cnr1), or GABA-A receptor subunit alpha-2^36^ (Figure 4E). In total, we identified 130 novel synaptic proteins that were significantly correlated with vGat expression and not previously recognized as synaptic by SynGO, for example, IgLON5, a cell-adhesion protein implicated in a specific neurodegenerative autoimmune disease.^37^

**Figure S5:**
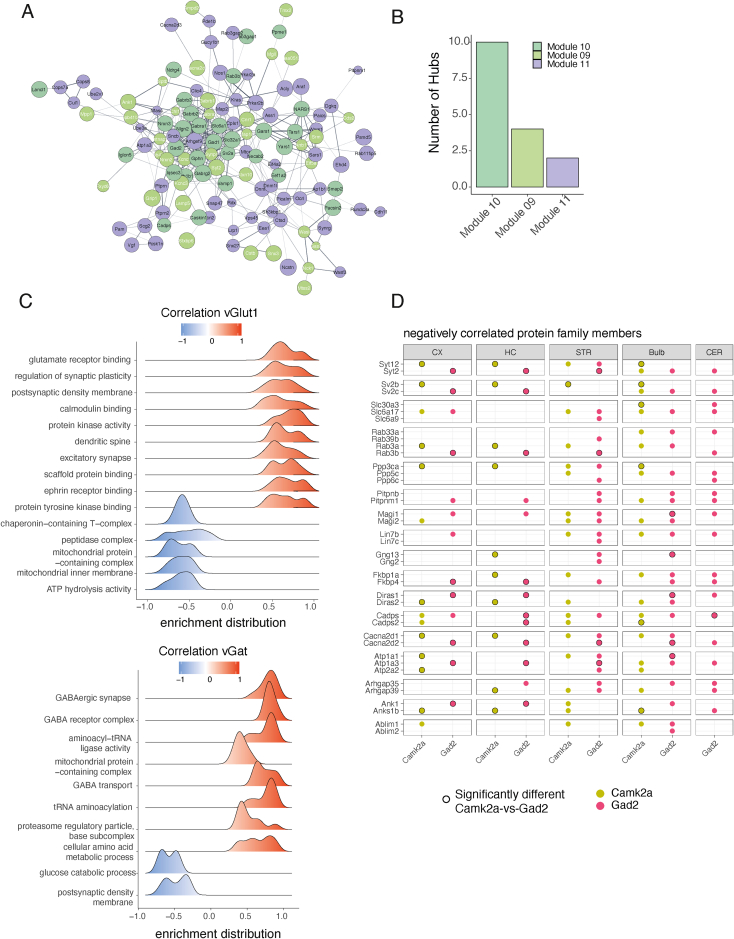
Validation of the protein-protein correlation network, related to Figure 4 (A) STRING network of proteins that are associated with the three modules that were significantly correlated with vGat (modules 9, 10, and 11). Edges are based on stringdb score >0.4. (B) Bar plot showing number of hubs from the network in (A) for each module. Hubs are defined as the top 10% most connected proteins in the network (centrality parameter “degree”). Modules 9, 10, and 11 represent the modules that were significantly correlated with vGat immunofluorescence. The core module (module 10) of the protein-protein correlation network also contains the most hubs in the STRING network from (A), and the auxiliary modules of the protein-protein correlation network (modules 9 and 11) account for fewer hubs in the STRING network. (C) Ridge plots for pathways enriched in vGlut1 and vGat protein communities. Ridge plots show enrichment distribution for core-enriched genes of selected significantly enriched terms. Gene set enrichment analysis (GSEA) was conducted, and proteins were ranked by their correlation with vGat (right) and vGlut1 (left) immunofluorescence. (D) Heatmap for negatively correlated protein pairs showing relative enrichment or de-enrichment across the 15 synaptic proteomes. Black around dots indicates a significant difference in the direct comparison of Camk2a vs. Gad2 proteomes. The protein correlations and p values of the negatively correlated protein pairs are in Table S3.

In order to validate the network topology of the GABAergic proteome, we constructed a protein-protein interaction network of the three inhibitory protein modules using a protein interaction database and found that the proteins from the core module (module 10) accounted for the majority of hubs at the center of the network (Figures S5A and S5B). In conclusion, we present a protein-protein correlation network that represents the diversity of the GABAergic synaptic proteome, revealing many novel synaptic proteins, and highlights core proteins that are strongly associated with vGat across synapses from different brain regions and cell types, as well as moderately associated proteins that may distinguish specific synapse subtypes.

The excitatory synapse protein community was well-correlated with VGlut1 fluorescence (Figure 4D); correspondingly, we found the VGlut1 protein was among the most connected nodes and there was no significant correlation with the VGlut2 protein. Overall, we detected many more proteins in the VGlut1+ network when compared to the vGat+ network. Presumably, this is due to the elaborate PSD complex and spine architecture present at excitatory synapses. Accordingly, we detected many proteins from established excitatory postsynaptic protein families within the VGlut1 protein community, including the Shank family (Shank1/2/3), the Camk family (Camk4/1d/2d/2b/v/2a), the Dlg family (Dlg1/2/3/4, Mpp2/3, and Dlgap1/2/3/4), the glutamate receptor subunits (Gria1/2/3/4, Grin1/2b, Grik2), the Lrr family (Lrrc7/57/4) and protein phosphatases (Pp2r5a/3ca/1cb/5c/1ca/3cb/3ra). We also identified many proteins not previously associated with glutamatergic synapses. For example, we found Fbxl16, an F-box protein of an E3-ubiquitin ligase with unclear synaptic function,^38^^,^^39^ exhibited the strongest correlation with VGlut1 expression across brain regions. In total, we identified 158 novel synaptic proteins that correlated significantly with VGlut1 expression and were not previously recognized as synaptic by SynGO.

We next asked whether there are pathways or biological functions enriched specifically at vGat vs. VGlut1 protein communities. To address this, we performed gene set enrichment analysis (GSEA) using the ranked protein correlations with vGat and VGlut1 immunofluorescence, respectively. As expected, we found significant enrichment for proteins associated with postsynaptic signaling pathways and dendritic spines within the VGlut1 community (Figures 4F and S5C). In the vGat protein community, we found significant enrichment for proteins from the GABA receptor complex, but also proteins with functions not previously recognized as enriched at inhibitory (vs. excitatory) synapses: aminoacyl-tRNA synthetases, 19S proteasome subunits, and mitochondrial proteins (Figures 4F and S5C).

We previously observed an opposite enrichment pattern of some proteins from the same family at excitatory and inhibitory synapses. To investigate this further, we conducted a comprehensive analysis and identified 22 protein pairs that exhibited significant negative correlations across the 15 synapse types (Figures 4G, 4H, and S5D; Table S3). The identified protein pairs spanned diverse functional categories, including exocytosis (Rab3a/b, Syt2/12, Sv2b/c), voltage-gated calcium channels (Cacna2d1/2), protein folding (Fkbp1a/p4), and ATPase activity (Atp1a1/3). Notably, our analysis also revealed proteins, such as the GTPases Diras1 (inhibitory) and Diras2 (excitatory), without prior synaptic annotation. These findings highlight their potential involvement in the same synaptic molecular processes to modulate synapse-type-specific characteristics. Taken together, the above analyses define a roadmap for how molecular systems-biology analysis of synaptic proteomes can be used to identify the key protein modules that underlie synaptic traits.

**Figure 5:**
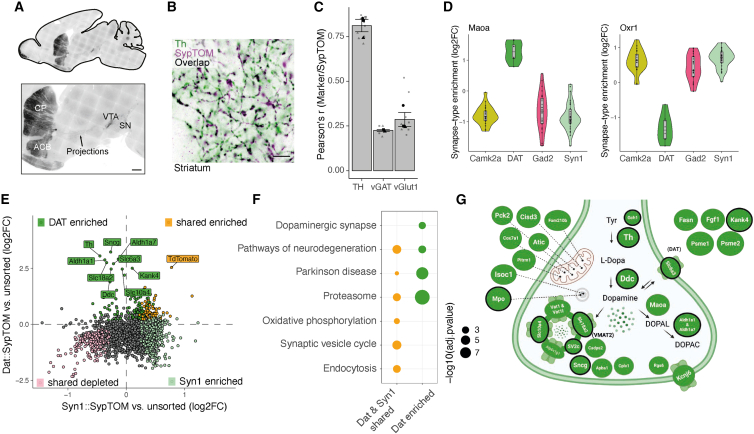
The dopaminergic synaptic proteome (A) Representative images of an immunostained brain section from a Dat::SypTOM mouse showing tdTomato present in the nigrostriatal pathway. Fluorescent signal was detected in the cell bodies of the ventral tegmental area (VTA), substantia nigra (SN), and their associated projections to striatal areas caudate putamen (CP) and nucleus accumbens (ACB). Scale bars, 500 μm. (B) Representative image depicting overlap between tyrosine hydroxylase (Th) immunoreactivity with tdTomato fluorescence in the striatum of a Dat::SypTOM mouse. Scale bars, 5 μm. (C) Analysis of data shown in (B) supplemented by correlation of tdTomato immunoreactivity with VGlut1 and vGat in the striatum of Dat::SypTOM mice. n = 2–4 animals, 2–4 images per mouse, error bars = the standard error of the mean. (D) Violin plots for two representative proteins showing the specific enrichment (amino oxidase A, Maoa, a marker for dopaminergic neurons) or depletion (oxidation resistance protein 1 [Oxr1]) in dopaminergic synaptic terminals compared with all other synapse types. (E) Scatter plot comparing the differential enrichment of proteins in the Dat+- and Syn1+-synaptosomes to their striatal control synaptosome precursor populations. Colors indicate proteins that were significantly enriched in Dat+-sorted synaptosomes (green), Syn1+-sorted synaptosomes (pale cyan), significant in both populations (orange), or significantly de-enriched in both (pale pink). (F) Dot plot of selected significantly enriched pathways (KEGG) of GSEA comparing exclusively dopaminergic synapse-enriched proteins with shared-enriched proteins between dopaminergic and all striatal synapses (Syn1+). (G) Scheme showing selected top-enriched proteins (Dat+ compared with unsorted controls or Syn1+) within the presynaptic terminal. The ten proteins with the highest fold-change difference compared with Syn1+ and unsorted controls are highlighted with a black border. See also Figure S6.

**Figure S6:**
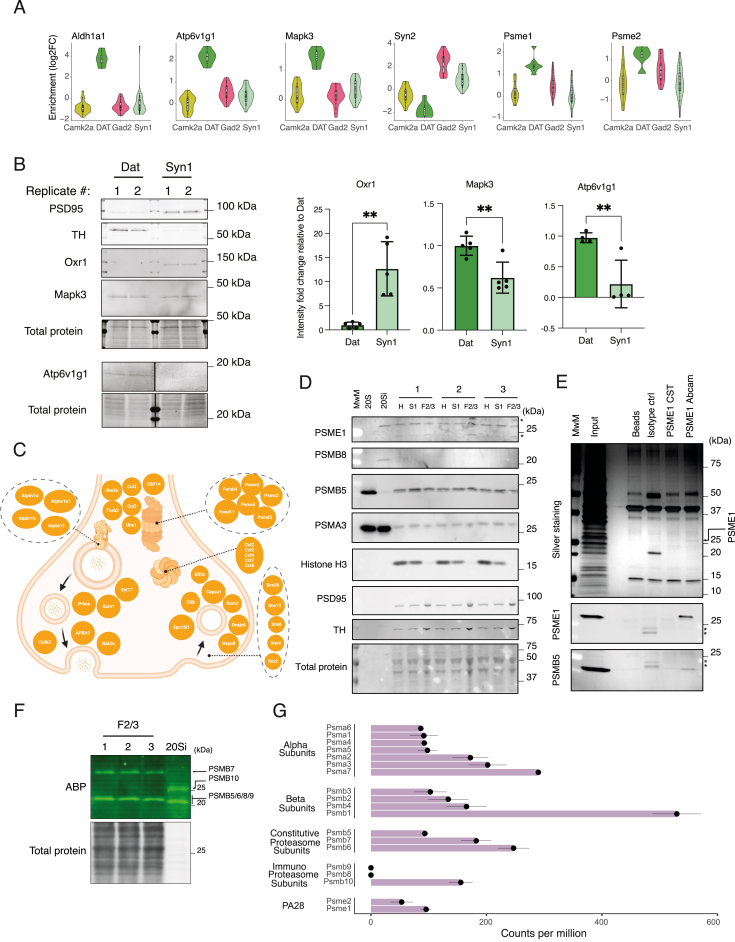
Analysis of the dopaminergic synaptic proteome, related to Figure 5 (A) Violin plots for representative proteins showing specific enrichment or specific depletion in dopaminergic synaptic terminals compared with all other synapse types. (B) Validation of Oxr1, Mapk3, and Atp6v1g1 enrichment or de-enrichment at Dat+ vs. Syn1+ synapses. Left: representative immunoblots of striatal Syn1+ and Dat+ synaptosomes. Each lane corresponds to 20 Mio sorted synaptosomes. Shown are two independent replicates per genotype. Right: histograms of the corresponding quantifications of the immunoblots. ^∗∗^p ≤ 0.01, unpaired t test, two-tailed; the data reported are mean and SD. n = 5 for Mapk3 and Oxr1; n = 4 for Atp6v1g1. (C) Scheme showing selected proteins that were enriched in Dat+ synapses and Syn1+ synapses within the presynaptic terminal. (D) SDS-PAGE and immunoblot of the different fractions of the synaptosome preparation (H, homogenate; S1, soluble fraction; F2/3, synaptosome fraction) from mouse striatum (n = 3 biological replicates). Although in the F2/3 fractions synaptic proteins PSD95 and Th were enriched, the nuclear protein histone H3 was not detectable. The constitutive proteasome subunits PSMA3 and PSMB5 as well as the PA28 regulatory particle subunit PSME1 were found across all fractions at comparable levels. By contrast, the immunoproteasome subunit PSMB8 was only detected in the purified 20Si proteasome sample. (E) Immunoblot of a PSME1 co-immunoprecipitation experiment using primary rat cortical neurons. The experiment shows that in rat cortical neurons PSME1 interacted with the constitutive 20S proteasome. (F) SDS-PAGE of mouse striatal F2/3 fractions and purified 20Si proteasome assayed for proteasome activity using an activity-based probe.^73^ Although activity corresponding to the PSMB10 subunit of the 20Si proteasome was visible in the purified sample, this was not the case in the F2/3 striatal fractions. Asterisks indicate non-specific bands. (G) Re-analysis of published single-cell RNA sequencing (scRNA-seq) data^36^ showing expression of proteasomal genes in midbrain dopaminergic neurons. mRNA for the constitutive proteasome subunits was detected, whereas mRNA of 2 of 3 immunoproteasome subunits was not detected.

Dopaminergic synapses from midbrain dopaminergic neurons that project to the STR are critically important for reward processing and movement control,^40^ and their degeneration is a hallmark of Parkinson’s disease pathophysiology.^41^ We conducted an in-depth analysis of the synaptic proteome of striatal dopaminergic terminals. To verify the expression of the presynaptic fluorophore in the Dat::SypTOM mice, we immunostained brain sections (Figure 5A) and found a prominent tdTomato signal in the STR and a high correlation with tyrosine hydroxylase (Th) immunoreactivity (Figures 5B and 5C). Comparing striatal Dat::SypTOM synaptosomes with unsorted control synaptosomes, we identified 267 significantly enriched proteins in the striatal dopaminergic proteome (Table S2A). To address specific vs. shared dopaminergic synaptic proteins, we identified proteins that were either enriched or de-enriched in striatal dopaminergic synapses as compared with the other 14 synapse types. Among the differentially enriched proteins were dopaminergic marker proteins, for example, Maoa and Aldh1ha1, but also proteins that have not been previously associated with dopaminergic terminals (Figures 5D and S6A). For example, we identified oxidation resistance protein 1 (Oxr1), as ubiquitously present in Camk2a, Gad2, and Syn1 synapse types but depleted from dopaminergic terminals (Figure 5D). Oxr1 controls sensitivity to oxidative stress^42^^,^^43^ and as such, the absence of Oxr1 in dopaminergic synapses might confer susceptibility to oxidative damage (e.g., during Parkinson’s disease). Another example is the mitogen-activated protein kinase Erk1 (Mapk3), which was specifically enriched at dopaminergic synapses (Figure S6A) and has been linked to Parkinson’s disease via multiple cellular processes.^44^ We validated these findings and the findings below with immunoblotting (Figure S6B). Next, we compared the striatal Dat::SypTOM proteome to the striatal Syn1::SypTOM proteome (Figure 5E) and performed GSEA comparing mutually enriched with Dat::SypTOM specific proteins (Figure 5F). Relative to striatal Syn1::SypTOM synapses, the Dat::SypTOM synaptic proteome was enriched in key proteins involved in dopamine biosynthesis, trafficking, and degradation (Figures 5E and 5G). The proteins that were significantly enriched in both groups included vATPases, proteasome subunits, and endocytic proteins (Figures 5F and S6C). Although we identified four vATPases within the shared group (Atp6v1a/h/f/e1), Atp6v1g1 was significantly enriched at dopaminergic synapses, indicating an association with dopaminergic synaptic vesicles (Figure 5G). We identified six proteasome subunits as shared and two proteasome subunits that constitute the modulatory immunoproteasome-associated PA28 complex (Psme1/2) as enriched at dopaminergic synapses. We validated the presence of the Psme1 protein and proteasome activity in striatal synaptosomes but found no evidence for the presence of immunoproteasome subunits, rather, Psme1 was associated with standard proteasome subunits (Figures S6D–S6G). The above data identify the synaptic proteome of striatal dopaminergic terminals and highlight similarities and specializations in synaptic proteome architecture.

**Figure S7:**
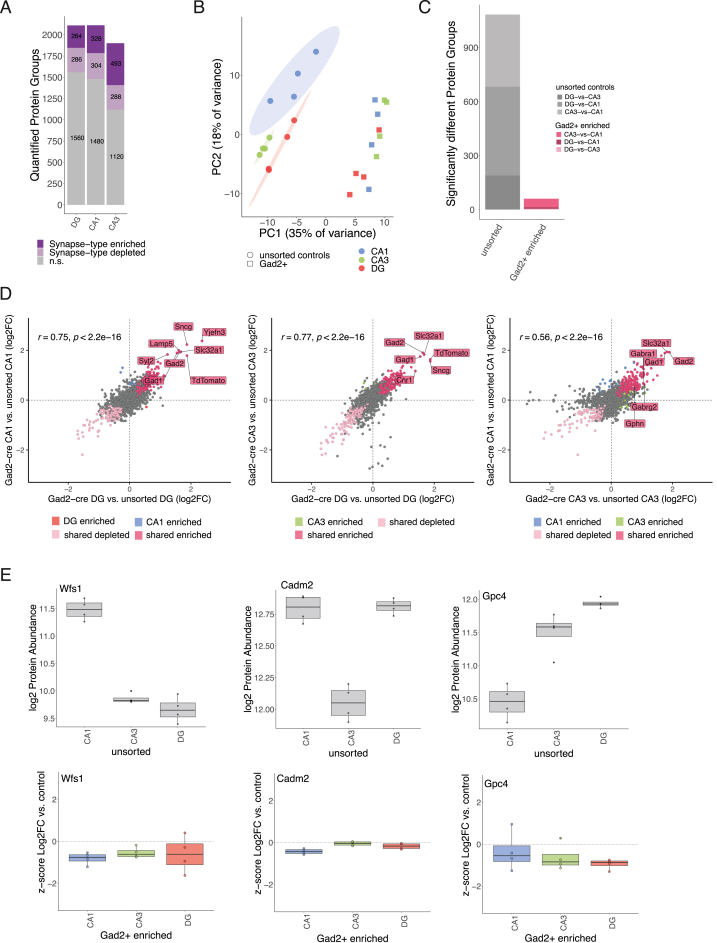
Inhibitory synaptic proteomes of the hippocampus, related to Figure 6 (A) Number of protein groups quantified for each hippocampal subfield interneuron synapse type. Shown are significantly enriched and de-enriched groups as well as protein groups that are not significantly different between the groups. (B) PCA of Gad2+-enriched and unsorted control samples from hippocampal subfields. Note distinct clustering of control samples and interspersed Gad2+-enriched samples, indicating global subfield-specific differences in proteome composition in crude synaptosome fractions but not Gad+-enriched fractions. (C) Bar plot showing significantly different protein groups for comparisons between unsorted controls or Gad2+-enriched fraction. (D) Correlation plots of Gad2+-enriched fractions vs. unsorted controls comparing different hippocampal formation subfields. Note the significant high correlation between all subfields and shared enrichment of inhibitory synapse marker proteins, indicating high similarity of Gad2+-enriched synaptic proteomes across hippocampal subfields. (E) Boxplots for some selected proteins that showed subfield-specific enrichment in unsorted control fractions (upper) and the absence of regulation of these proteins in Gad2+-enriched fractions.

We next asked whether our approach can be used to study synapse types originating from small subfields or rare cell types. To analyze subfields, we microdissected the main hippocampal regions CA1, CA3, and the dentate gyrus (DG) from a single Gad2::SypTOM mouse (per replicate) and quantified the inhibitory synaptic proteomes. Despite the small amount of starting material, we quantified more than 2,000 protein groups and discovered 264 (DG), 328 (CA1), and 493 (CA3) Gad2-enriched proteins in the subfield-specific inhibitory proteomes (Figure S7A; Table S4). Although the unsorted control samples showed subfield-specific clustering and enrichment of known marker proteins, the inhibitory proteomes were highly correlated and had only few differentially enriched proteins (Figures S7B–S7E).

**Figure 6:**
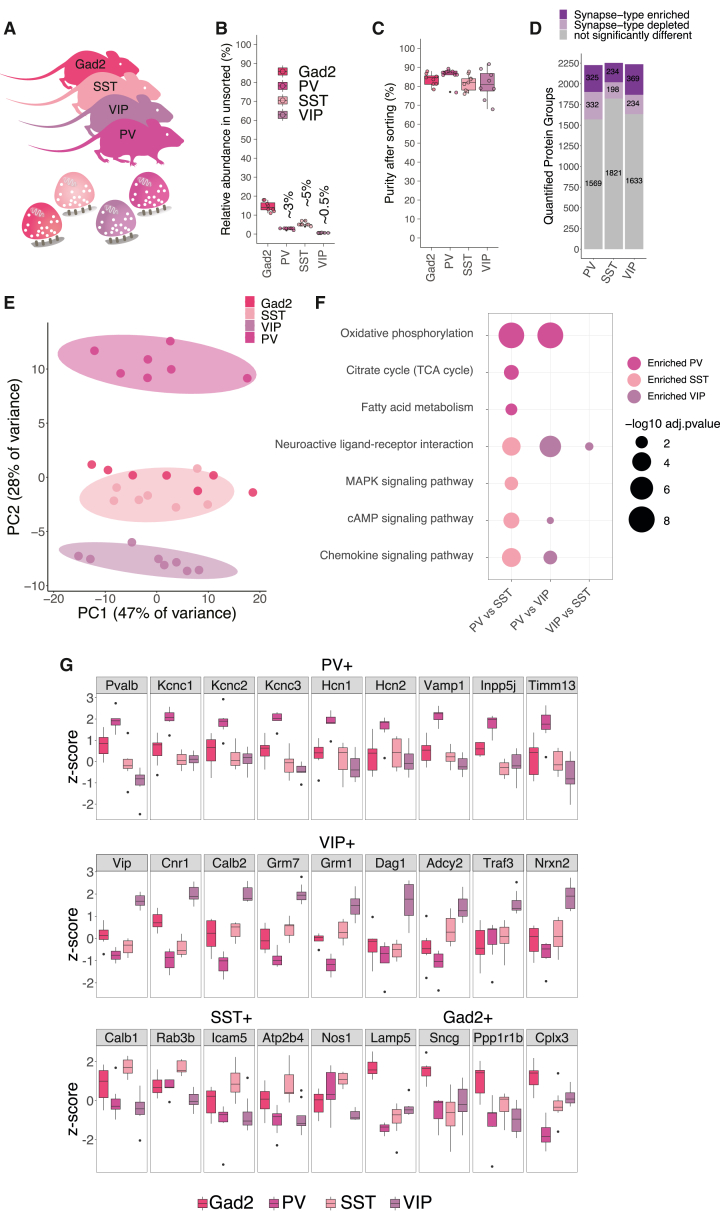
Proteomic diversity of Gad2, parvalbumin, somatostatin, and vasointestinal active peptide synapses (A) Scheme showing the different mouse lines from which cortical synaptosomes were prepared. (B) Plot indicating the relative abundance of each fluorescently labeled synapse type in the crude cortical synaptosome fraction. (C) Plot indicating the purity of each fluorescently labeled cortical interneuron synaptosome type after FASS. For all types, the average purity exceeded 80%. Purity is assessed by re-analysis of the sorted fraction by synaptosome flow cytometry. (D) Number of protein groups quantified for each cortical interneuron synapse subtype. Shown are significantly enriched and de-enriched groups as well as protein groups that are not significantly different between the groups. (E) PCA of synaptic proteomes from cortical inhibitory subtypes. (F) Dot plot of selected significantly enriched pathways (KEGG) of GSEA comparing cortical interneuron types with one another. Analysis is based on protein lists ranked by log_2_FC of the synapse types indicated on the x axis. (G) Boxplots for representative proteins that show specific enrichment in the indicated cortical interneuron subtype. Boxplot indicates the median, 25th and 75th percentiles. See also Figures S7 and S8.

**Figure S8:**
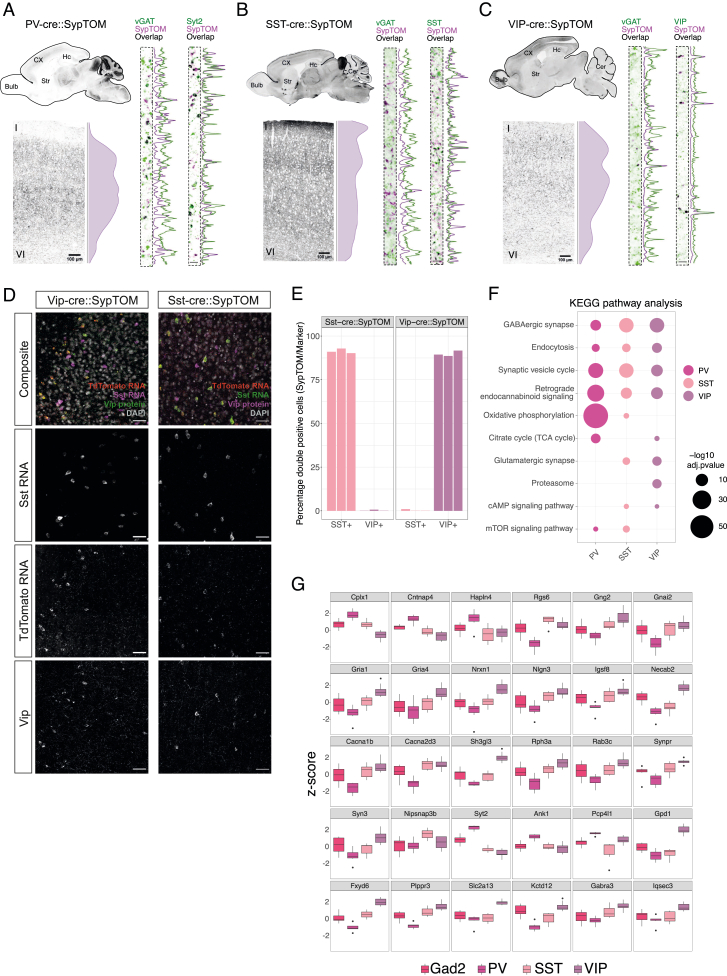
Supplementary analysis for cortical interneuron subtype proteomes, related to Figure 6 (A) Left: sagittal overview of SypTOM expression in a PV::SypTOM mouse and below an overview of SypTOM expression throughout the cortex of a PV::SypTOM mouse. The relative density of the SypTOM signal across the different cortical layers is plotted on the side. This analysis provides an overview of PV+ synapse abundance across the cortical layers. Right: representative image of a immunofluorescent co-staining for the inhibitory synapse marker solute carrier family 32 member 1 (vGat) and the synaptotagmin-2 (Syt2), a marker for synapses formed by PV neurons,^35^ both in green, and of SypTOM expression, in purple, in the CX of a PV::SypTOM mouse. Overlap is depicted in black; SypTOM overlaps with Syt2 and partially with vGat, which is further illustrated by maximum normalized fluorescent intensity line plots. (B) Same as (A) but for the SST::SypTOM mouse and the SST-neuron marker protein somatostatin. (C) Same as (A) but for the VIP::SypTOM mouse and the VIP-neuron marker protein VIP peptides. (D) Validation of labeling specificity of SST::SypTOM and VIP::SypTOM mice. Representative images for both mouse lines showing RNA fluorescence *in situ* hybridization (FISH) signal for sst and TdTomato mRNA, immunoreactivity for the Vip protein, and TdTomato fluorescence. Scale bars, 50 μm. (E) Quantification of the experiment shown in (D). Both mouse lines show a high labeling specificity for their respective cell type. Around 90% of the cells that express TdTomato in SST::SypTOM mice express sst mRNA, and very few show immunoreactivity for Vip. The inverse is true for VIP::SypTOM mice. n = 3, each bar represents one animal. (F) KEGG pathway enrichment analysis of cortical interneuron proteomes. (G) Boxplots for indicated representative proteins show specific enrichment in the indicated cortical interneuron subtypes. Boxplots indicate the median, 25th and 75th percentiles of standardized (*Z* score) protein abundances. Black dots, outliers.

The neurons that use the neurotransmitter GABA exhibit morphological diversity and differ in the location of their cell bodies and synapses on other cells,^45^ as well as their transcriptomic profiles, connectivity patterns, and firing properties.^46^^,^^47^^,^^48^ Using Cre-driver lines for the main subclasses, parvalbumin (PV), somatostatin (SST), and vasoactive intestinal peptide (VIP) neurons, we targeted synapses from these cortical GABAergic neuron subclasses^25^ (Figure 6A) and compared them to the cortical Gad2::SypTOM synaptic proteome. We verified the co-localization of tdTomato signal with subtype-specific markers (Figures S8A–S8C) and validated the specificity of SST- and VIP-Cre-driver lines (Figures S8D and S8E). We note that cortical interneurons are very sparse, making their synaptic proteomes very challenging to study. Indeed, we found that VIP+ synaptosomes represented just ∼0.5% of all particles in a cortical synaptosome fraction, whereas PV+ and SST+ synaptosomes represented approximately 3% and 5% (Figure 6B). After sorting, we achieved greater than 80% purity for all 3 cortical inhibitory synapse types (Figure 6C). With an optimized sample processing protocol, we quantified >2,500 protein groups in total and identified almost 600 unique proteins enriched in at least one cortical interneuron subtype. We found distinct synaptic proteomes for the three subtypes with 325, 234, and 369 significantly enriched proteins detected, respectively, for PV+, SST+, and VIP+ synapses (Figure 6D; Table S5A). All proteomes were significantly enriched with GABAergic synaptic proteins (Figure S8F). The three inhibitory synaptic proteomes were clearly separated in a PCA (Figure 6E) and showed a type-specific signature in their proteome composition (Figure 6F). The union Gad2 proteome was closest to the most abundant type SST+ in PCA space. We searched for canonical markers of each inhibitory synapse type and found, as predicted, the expected enrichment for VIP, PV, and Calbindin proteins in the VIP+, PV+, and SST+ synaptic proteomes (Figure 6G). The remaining markers, Lamp5 and Scng, distinguished a 4th and 5th type of inhibitory neuron^25^^,^^45^ and, appropriately, were most enriched in the Gad2+ cortical synaptosomes (Figure 6G).

We next asked whether the developmental origin of the cell type is reflected in the synaptic proteome. Although PV and SST neurons arise from the medial ganglionic eminence (MGE), VIP neurons originate from the caudal ganglionic eminence (CGE).^49^ Previous studies showed that transcriptomes of cortical inhibitory neurons^36^^,^^50^^,^^51^ cluster according to their progenitor domain. By contrast, a PCA revealed that the SST+ and VIP+ synaptic proteomes cluster closer together than the SST+ and PV+ proteomes (Figure 6E). Consistent with this, we identified only 33 proteins that were significantly different between the SST+ and VIP+, but 128 and 318 between PV+ and SST+ or VIP+, respectively (Tables S5B–S5D). These analyses indicate that cortical inhibitory synaptic proteomes are predominantly shaped by other factors than the developmental origin of their presynaptic cell type.

Can we identify proteins that relate to the functional differences observed in these three inhibitory synapse types? PV+ neurons are characterized by fast spiking and corresponding high energy demands.^52^ We found that the voltage-gated potassium channels Kcnc1, Kcnc2, and Kcnc3 (Kv3.1/2/3) were among the most enriched PV+ synaptic proteins (Figure 6G), potentially explaining the ability of PV cells to accommodate high firing rates.^53^ We also found Hcn1 and Hcn2 were highly enriched in the PV+ synaptic proteome (Figure 6G). The PV+ proteome was also significantly enriched in mitochondrial proteins involved in oxidative phosphorylation (Figure 6F). Additionally, we detected a number of synaptic vesicle-associated proteins (such as Syt2, Vamp1, or Cplx1) and the cell-adhesion proteins Cntnap4^54^ and Hapln4 as specifically enriched at PV+ synaptic proteomes (Figures 6G and S8G; Table S5).

Cortical VIP neurons are recognized for their inhibitory control over SST and PV interneurons, forming a crucial component of cortical disinhibitory circuits. In comparison to the cortical PV+ proteome, the VIP+ synaptic proteome was enriched for neuroactive ligand-receptor interactions and downstream signaling molecules (Figures 6F and 6G). One of the most enriched proteins was Cnr1, and many proteins involved in downstream G-protein signaling were enriched as well, including Rgs6, Kcd12, Adcy2, Gng2, and Gnai2 (Figure 6G; Table S5). We also detected a strong enrichment of glutamate G-protein coupled receptors, the metabotropic glutamate receptors (excitatory Grm1 and inhibitory Grm7), and, to a lesser extent, the ionotropic glutamate receptor subunits Grik2 and Gria1/4 but not Gria2/3 (Figures 6G and S8G; Table S5). Furthermore, we found a number of cell-adhesion proteins specifically enriched at cortical VIP+ synapses (Nrxn1, Nrxn2, Nlgn3, Dag1, and Igsf8), as well as calcium-binding proteins (Calb2 and Necab2), calcium channels (Cacna1b and Cacna2d3), and synaptic-vesicle-associated proteins (Sh3gl3, Rph3a, Rab3c, Synpr, and Syn3) (Figures 6G and S8G; Table S5). In contrast to PV+ and VIP+, there were few proteins (Calb1, Rab3b, Icam5, Nipsnap3b, Atp2b4, and Nos1) that distinguished SST+ synaptic proteomes (Figure 6G). This reflects the current view that SST neurons represent a diverse group with substantial differences in morphology and physiology.^55^ Finally, we identified a few proteins (Ppp1r1b and Cplx3) that were enriched in the overall Gad2 synaptic proteome over the three inhibitory subtypes, presumably because they are specific for one of the other main cortical interneuron subtypes characterized by Lamp5 or Scng expression (Figure 6G). Overall, we identified ∼600 unique proteins that define the cortical interneuron synaptic proteome, allocated them to the three main subclasses, and highlighted specialized groups of proteins that relate to their established functional properties.

## Discussion

Although there is evidence for substantial structural and functional diversity of synapses,^4^^,^^56^ the underlying diversity in synaptic molecular architecture is much less understood. A detailed understanding of synapse proteome diversity allows us to link the molecular architecture of the synapse to structure and function. Here, we optimized FASS^22^ in combination with MS for system-wide analysis of the proteomic landscape of synaptic diversity across 18 distinct synapse types defined by cell type and brain region. We found that the cell type from which the synapses originate explains more of the observed variance than the brain region. Across the brain areas, the Bulb and CER were the most distinct compared with the other brain areas. These regions may exhibit unique molecular profiles that reflect their evolutionary context and developmental origin and potentially their distinct roles in sensory processing (Bulb) and motor coordination (CER). With these data, we now define a common core of synaptic proteins, shared across synapse types, that includes vATPases and synaptic vesicle endocytosis proteins; by contrast, we identified the presynaptic active zone, exocytosis machinery, transsynaptic, and postsynaptic elements as hotspots for synaptic proteome specialization. Our analysis also revealed the polarized enrichment of individual proteins from the same family across synapse types, suggesting a means by which functional differences in these protein family members can modulate the same synaptic molecular process to establish synapse-type-specific characteristics.

Using a “guilt-by-association approach” (the WGCNA^33^), we found that synaptic proteins form communities that correlate with vGat and VGlut1 expression. Intriguingly, we discovered many proteins at the core of the vGat and VGlut1 protein communities that were not previously recognized as synaptic. The vGat protein community further revealed an intriguing enrichment of unanticipated functional protein groups: we identified proteasome subunits, mitochondrial proteins as well as tRNA synthetases preferentially enriched over the VGlut1 synaptic community. The presence of mitochondrial proteins in the vGat community presumably relates to increased energy demands. Although moonlighting functions have been ascribed to tRNA synthetases,^57^ it is possible that the tRNA synthetases scavenge and charge amino acids that originate from local proteasomal degradation.

We also provide an in-depth analysis of striatal dopaminergic synapses, revealing 267 proteins significantly enriched at dopaminergic terminals, which is an almost 5-fold greater depth compared with a recent proteome analysis of dopaminergic terminals.^19^ We compared the dopaminergic proteome to the other 14 synaptic proteomes and identified the absence of Oxr1, a protein that protects from oxidative damage and might render dopaminergic neurons particularly susceptible to oxidative stress.^43^^,^^58^ Finally, we identified ∼600 unique proteins that define the synaptic proteomes of the main cortical interneuron subclasses. In contrast to the transcriptomes of cortical interneurons, we find that the synaptic proteomes do not cluster according to their progenitor domain, indicating that synaptic proteomes are shaped by factors other than the developmental origin of their presynaptic cell type. We reveal type-specific signatures in the cortical interneuron proteomes that relate to their established functional properties. For PV+ synapses, characterized by high firing frequencies, we identified a specific enrichment of mitochondrial proteins, voltage-gated potassium channels, and hyperpolarization-activated cyclic nucleotide-gated ion (HCN) channels. We detected the differential distribution of individual CAMs (e.g., Nrxn1, Nrxn2, Cntnap4, Hapln4, and Icam5), suggesting a role in the distinct connectivity patterns observed for the interneuron subtypes.

Our resource of synaptic proteomes departs from previous studies in many respects. First, we used FASS^22^ because it enables the analysis of synaptic proteomes originating from *in vivo* brain structures and covers all synaptic compartments, including pre-, post-, and transsynaptic proteins. Second, we identified the proteome of relatively scarce synapse types not previously amenable to purification (e.g., the VIP+ synaptic proteome, representing <1% of the total cortical synapse pool). Third, we included many biological replicates per synapse type (>5), increasing the proteome depth to define synapse-enriched proteins in a purely data-driven fashion using linear-mixed effects models,^59^ without the requirement for external lists or prior knowledge. Importantly, we used a paired-sample experimental design where every sorted sample was accompanied by an unsorted control, and synapse-enriched proteins were determined by quantitative enrichment. Fourth, we compared proteome diversity across 15 synapse types, whereas all previous studies were limited to one or two synapse types.^7^^,^^9^^,^^11^^,^^14^^,^^15^^,^^16^^,^^17^^,^^19^^,^^22^^,^^24^^,^^60^ Finally, our resource enables a direct comparison of different synapse types, in contrast to comparisons between synapses and other subcellular compartments like the soma.^60^

We anticipate that multi-omic analysis of synapses will be conducted using an analogous experimental strategy, investigating the synaptic diversity of other biomolecules like glycans, lipids, or RNA. In addition, the future integration of synaptic proteomes with local transcriptomes can delineate the roles of RNA localization and local translation in synaptic proteome diversity.^61^^,^^62^^,^^63^^,^^64^ On the cellular level, experiments that use methods such as the Cre/lox system^65^ to target a defined cell type to investigate function can now be combined with synapse subtype-specific proteome information and thereby connect different levels of organization. For example, one could probe how a particular phenotype, disease model, behavioral paradigm, or cellular manipulation differentially affects the synaptic proteomes of the synapse subtypes of interest.

### Limitations of the study

This study combines transgenic reporter mice with FASS and MS-based proteomics, and each aspect has inherent limitations. The reporter animals limit the cell-type-specific synaptic proteomes that can be targeted and dictate the resulting homogeneity. FASS is based on biochemical synaptosome purification and may be biased toward synapses that readily form synaptosomes of the described size and density while limiting the analysis of synapses with anomalous sizes or shapes. Furthermore, synaptosomes are sorted using a presynaptic marker protein, and not all sorted synaptosomes contain an intact postsynaptic compartment; as such, there may be a bias against postsynaptic proteins. MS is biased in its ability to quantify peptides by the individual peptides' biophysical properties that influence ionization efficiency and transfer to the gas phase. We identified enriched synaptic proteins from fluorescently labeled synaptosomes by comparing them to their unsorted precursor synaptosome population, which includes the labeled synapse population of interest as well as unlabeled synapses. As such, proteins that we identify as enriched at synapse type A over type B or correlated with vGat or VGlut1 might be present in neuronal dendrites, axons, or somata to varying degrees. The observed enrichments or de-enrichments are thus a function of protein abundance and specific subcellular targeting or exclusion of proteins from synapses. For example, proteins that showed cell-type-specific expression but broad localization throughout the cell, like the PV protein, were identified as specifically enriched in PV+ synapses when compared with other synapse types. By contrast, specific recruitment or synaptic exclusion could lead to synaptic enrichment or de-enrichment despite comparable average protein abundance in different neuron types. For example, Oxr1 is specifically enriched at many synapse types but depleted from dopaminergic terminals; however, the corresponding mRNA was found at comparable levels throughout many neuron types, including midbrain dopaminergic neurons and GABAergic neurons in the STR.^36^

## STAR★Methods

### Key resources table

**Table:** 

REAGENT or RESOURCE	SOURCE	IDENTIFIER
**Antibodies**		

anti-vGAT	SYSY	Cat#131004 and Cat#131002; RRID:AB_887873 and RRID:AB_887871
anti-vGlut1	SYSY	Cat#135304 and Cat#135303; RRID:AB_887878 and RRID:AB_887875
anti-TH	SYSY	Cat#213104; RRID:AB_2619897
anti-VIP	Thermo Fisher	Cat#PA5-78224; RRID:AB_2736784
anti-SST	Thermo Fisher	Cat#PA5-85759; RRID:AB_2792896
anti-Syt2	SYSY	Cat#105223; RRID:AB_2619770
anti-NeuN	Abcam	Cat#Ab177487; RRID:AB_2532109
anti-PSD95	Thermo Fisher	Cat#MA1-046; RRID:AB_2092361
anti-PSD95	Abcam	Cat#ab2723; RRID:AB_303248
anti-Mbp	Abcam	Cat#Ab62631; RRID:AB_956157
anti-GFAP	Abcam	Cat#Ab7260; RRID:AB_305808
anti-Syn	SYSY	Cat#106002; RRID:AB_887804
anti-SYPH	Sigma	Cat#S5768; RRID:AB_477523
anti-Gad1/2	Enzo	Cat#ADI-MSA-225-E; RRID:AB_2039129
anti-Syt12	SYSY	Cat#299003; RRID:AB_2620044
anti-Proton ATPase	SYSY	Cat#109003; RRID:AB_10889596
anti-Complexin-1/2	SYSY	Cat#122102; RRID:AB_887708
anti-Stx1a	SYSY	Cat#110111; RRID:AB_887848
anti-Stx1b	SYSY	Cat#110403; RRID:AB_887900
anti-Syt2	SYSY	Cat#105223; RRID:AB_2619770
anti-Rab3c	SYSY	Cat#107203; RRID:AB_887771
anti-Vamp1	SYSY	Cat#104002; RRID:AB_887807
anti-Vamp2	SYSY	Cat#104202; RRID:AB_887810
anti-Oxr1	Abcam	Cat#ab251774
anti-Mapk3	CST	Cat#9102; RRID:AB_330744
anti-Atp6v1g1	ProteinTech	Cat#16143-1-AP; RRID:AB_2062686
anti-PSMA3	Enzo	Cat#BML-PW8110-0100
anti-PSMB8	Enzo	Cat#BML-PW8845-0100
anti-PSMB5	CST	Cat#12919; RRID:AB_2798061
anti-PSME1	Abcam	Cat#ab155091; RRID:AB_2801483
anti-Histone H3	Abcam	Cat#ab1791; RRID:AB_302613
anti-Gephyrin	SYSY	Cat#147011; RRID:AB_887717

**Chemicals, peptides, and recombinant proteins**		

Percoll	Cytiva	Cat#GE17-0891-01
Me4BodipyFL-Ahx3Leu3VS	Bio-Techne GmbH	Cat#I-190-050
20S proteasome	LifeSensors	Cat#PS020
20Si proteasome	Enzo	Cat#BML-PW9645-0050
FM4-64	Thermo Fisher	Cat#T13320
Protease Inhibitor Cocktail Set III, EDTA-Free	Merck	Cat#539134
viewRNA vGlut1	Thermo Fisher	Cat#VB1-15833-VC
viewRNA Gad2	Thermo Fisher	Cat#VB6-17621-VC
viewRNA TdTomato	Thermo Fisher	Cat#VF1-14985, VF6-13925
Sequencing Grade Modified Trypsin	Promega	Cat#V5111
Lys-C, Mass Spec Grade	Promega	Cat#VA1170
Triethylammonium bicarbonate buffer	Merck	Cat#T7408

**Critical commercial assays**		

Alexa Fluor 488 Tyramid SuperBoost Kit	Invitrogen	Cat#B40941
Precision red advanced protein assay	Cytoskeleton, Inc.	Cat#ADV02-A
Pierce BCA Protein Assay Kit	Thermo Fisher	Cat#23225
Revert 700 total protein stain	LI-COR	Cat#926-11011
Ponceau stain	CST	Cat#59803
ZipTip C18	Merck	Cat#ZTC18S

**Deposited data**		

Mass spectrometry data	This study	ProteomeXchange: PXD039946
scRNA sequencing data	Zeisel et al.^36^	http://mousebrain.org/
SynGO database	Koopmans et al.^5^	https://syngoportal.org/
CORUM database	Giurgiu et al.^34^	http://mips.helmholtz-muenchen.de/corum/
STRING database	Szklarczyk et al.^31^	https://string-db.org/

**Experimental models: Organisms/strains**		

Mouse: SypTOM (Ai34D): B6;129S-Gt(ROSA)26Sortm34.1(CAG-Syp/tdTomato)Hze/J	The Jackson Laboratory	RRID:IMSR_JAX:012570
Mouse: Camk2a-cre: B6.Cg-Tg(Camk2a-cre)T29-1Stl/J	The Jackson Laboratory	RRID:IMSR_JAX:005359
Mouse: Gad2-Cre: STOCK Gad2tm2(cre)Zjh/J	The Jackson Laboratory	RRID:IMSR_JAX:010802
Mouse: Syn1-cre: B6.Cg-Tg(Syn1-cre)671Jxm/J	The Jackson Laboratory	RRID:IMSR_JAX:003966
Mouse: DAT-IRES-cre: B6.SJL-Slc6a3tm1.1(cre)Bkmn/J	The Jackson Laboratory	RRID:IMSR_JAX:006660
Mouse: PV-cre: B6;129P2-Pvalbtm1(cre)Arbr/J	The Jackson Laboratory	RRID:IMSR_JAX:008069
Mouse: Sst-IRES-Cre: STOCK Ssttm2.1(cre)Zjh/J	The Jackson Laboratory	RRID:IMSR_JAX:013044
Mouse: Vip-IRES-cre: STOCK Viptm1(cre)Zjh/J	The Jackson Laboratory	RRID:IMSR_JAX:010908

**Software and algorithms**		

Proteome Discoverer 2.4	ThermoFisher	N/A
Spectronaut 16	Biognosys	N/A
FlowJo 10	BD	N/A
MSstats 4	Choi et al.^59^	https://msstats.org/
WGCNA	Langfelder and Horvath^33^	https://horvath.genetics.ucla.edu/html/CoexpressionNetwork/Rpackages/WGCNA/
Cytoscape	Shannon et al.^66^	https://cytoscape.org/
Prism 9	Graphpad	N/A
clusterprofiler	Yu et al.^67^	https://guangchuangyu.github.io/software/clusterProfiler/
SynaptosomesMacro	Paget-Blanc et al.^19^	https://github.com/fabricecordelieres/IJ-Toolset_SynaptosomesMacro

**Other**		

Resource webpage for data viewing	This paper	http://SynDive.org/

### Resource availability

#### Lead contact

Further information and requests for resources and reagents should be directed to and will be fulfilled by the lead contact, Erin Schuman (erin.schuman@brain.mpg.de).

#### Materials availability

This study did not generate new unique reagents.

### Experimental model and study participant details

#### Animals

The housing and sacrificing procedures involving animal treatment and care were conducted in conformity with the institutional guidelines that are in compliance with national and international laws and policies (DIRECTIVE 2010/63/EU; German animal welfare law; FELASA guidelines). The animals were euthanized according to annex 2 of § 2 Abs. 2 Tierschutz-Versuchstier-Verordnung. Animal numbers were reported to the local authority (Regierungspräsidium Darmstadt). The following Cre-driver lines were crossed with the SypTOM (JAX strain #: 012570, Ai34D) mouse line: Camk2a-cre^69^ (JAX strain #: 005359), Gad2-cre^25^ (JAX strain #: 010802), Syn1-cre^70^ (JAX strain #: 003966), Dat-cre^71^ (JAX strain #: 006660), PV-cre^72^ (JAX strain #: 008069), SST-cre^25^ (JAX strain #: 013044) and VIP-cre^25^ (JAX strain #: 010908). Animals were housed under a 12 h light/dark cycle, and provided with food and water ad libitum. Analyses were performed on adult animals aged 8-13 weeks with a median age of 10 weeks. The sex of the animals was mixed and balanced within conditions.

### Method details

#### Histology

Mice were transcardially perfused with 4% paraformaldehyde (PFA) in PBS and brains were post-fixed overnight (ON) in 4% PFA at 4°C. Sagittal sections (50μm) were cut using a Leica vibratome (VT1200S) and washed in PBS. Vibratome sections were blocked and permeabilized by incubation in 5% goat serum 0.5% Triton-x in PBS (blocking buffer) at RT for 4 hours. Primary antibody incubation was performed ON at 4°C on a rocker in the blocking buffer. The following day, sections were washed in PBS and incubated with a fluorescently labeled secondary antibody ON at 4 °C. Slices were washed in PBS, rinsed in ddH2O and air dried on SuperFrost Plus glass slides (Fisher Scientific). Aqua-Poly/Mount (Polysciences) was used for mounting.

For single molecule fluorescent *in situ* hybridization experiments (viewRNA, Thermo Fisher), mice were perfused and postfixed with 4% PFA, 4% sucrose in PBS for 1 hour at room temperature (RT). Cryoprotection was performed by incubating ON in 15% and subsequently 30% sucrose in RNAse free PBS at 4 °C. Sagittal sections (40μm) were cut using a Zeiss HYRAX S50 (at -25°C) and washed in PBS. Sections were post-fixed at RT in a 4% PFA solution (4% paraformaldehyde, 5.4% Glucose, 0.01 M sodium metaperiodate, in lysine-phosphate buffer) for 10 minutes, washed in RNAse free PBS and permeabilized using a detergent solution (viewRNA) for 20 minutes at RT. Probe hybridization was performed in the hybridization buffer at 40°C ON. The following day, sections were rinsed in the wash buffer and subjected sequentially to pre-amp DNA, amp DNA and label probe oligonucleotides in their respective buffers for 1 hour at 40°C with washes in the wash buffer at RT in between. After washing in PBS, sections were blocked in blocking buffer (4% goat serum 0.5% Triton-X in PBS). Primary antibody staining was performed ON in blocking buffer at 4°C. After washing in PBS, secondary antibody incubation was carried out at RT for 2 hours in blocking buffer. Sections were washed, counterstained with DAPI (1 μg/ml, Thermo Fisher) in PBS for 3 minutes, washed in PBS and mounted as described above. The following primary antibodies and corresponding dilution factors were used: anti-vGAT (SYSY, 131004 – gp, 1:5000), anti-vGAT (SYSY, 131002, rb, 1:5000), anti-vGlut1 (SYSY, 135304, gp, 1:5000), anti-vGlut1 (SYSY, 135303, rb, 1:5000), anti-TH (SYSY, 213104, gp 1:500), anti-VIP (Thermo Fisher, PA5-78224, rb, 1:500), anti-SST (Thermo Fisher, PA5-85759, rb, 1:500), anti-Syt2 (SYSY, 105223, rb, 1:500), anti-NeuN (Abcam, Ab177487, rb, 1:1000). The following FISH probes were used at 1:100 dilution: TdTomato (viewRNA, VF1-14985 or VF6-13925, Alexa Fluor 647 or Alexa Fluor 750), Sst (viewRNA,VB1-14821-VC or VB4-3112424-VC, Alexa Fluor 750 and Alex Fluor 647). Images were acquired using a Zeiss confocal microscope (LSM-880 or LSM-980) using 63X or 40X oil-immersion objectives (NA 1.4).

#### Synaptosome preparation

Synaptosomes were prepared as described in Westmark et al.^27^ Briefly, animals were sacrificed by decapitation, the brain regions of interest were dissected on ice and subsequently homogenized in gradient medium (GM; 0.25 M sucrose, 5mM Tris-HCl, 0.1mM EDTA supplemented with Calbiochem Protease inhibitor cocktail III) using a glass dounce homogenizer. The homogenate was centrifuged for ten minutes at 4°C at 1’000g. The supernatant (S1) was layered onto a Percoll density gradient with 23%, 10% and 3% Percoll in the GM buffer. The gradient was centrifuged for 5min at 32’500g at 4°C with maximum acceleration and minimum deceleration using a Beckman Coulter JA-25.50 rotor in an Avanti J-26S XPI centrifuge (both from Beckman Coulter). The resulting bands are labeled, from top to bottom, F0, F1, F2/3, F4. Bands were retrieved and directly processed (for EM analysis) or stored at -20°C. For proteasome activity measurements, F2/3 fractions were pipetted onto glass fiber filters using a syringe and stored at -80 °C until further processing. For immunoblotting, synaptosomes were lysed in a detergent buffer (8M urea, 10% SDS, 10% Sodium deoxycholate 5% Triton-X in water; 1 part detergent buffer and 5 parts synaptosome fraction) at 75°C for 5 minutes. BCA assay (Thermo Fisher) was used to approximate protein concentrations of the different fractions.

For synaptosomes of hippocampal subregions mouse brains were dissected on ice and coronal sections of 300 μm thickness were cut on a vibratome (Leica) in oxygenated slush ice prepared from frozen ACSF-sucrose (87 mM NaCl, 25 mM NaHCO_3_, 1.25 mM NaH_2_PO_4_, 2.5 mM KCl, 10 mM Glucose, 75 mM Sucrose, 0.5 mM CaCl_2_, 7 mM MgCl_2_). Hippocampal subregions were microdissected manually under a microscope with cooled stage from individually transferred sections and immediately collected in a Dounce homogenizer with 1 ml of GM buffer on ice. The tissue samples from each brain were processed as described above.

#### Proteasome activity assay

After fraction collection on filters (see above and below) proteasome activity was assayed by incubation of filters with HR buffer (50 mM Tris–HCl pH 7.4, 5 mM MgCl2, 250 mM sucrose)^73^ freshly supplemented with 1 mM DTT, 2 mM ATP and 1 μM Me4BodipyFL-Ahx3Leu3VS (Bio-Techne GmbH, I-190-050) for 1h at 37°C.^73^ To assay proteasome activity of 20S (LifeSensors, PS020) and 20Si (Enzo, BML-PW9645-0050) purified samples, 1 μg of protein was incubated in HR buffer complete with 1 μM Me4BodipyFL-Ahx3Leu3VS for 1h at 37°C. Sample protein concentration was measured with the Precision Red advanced protein assay (Cytoskeleton, Inc., ADV02-A).

#### SDS-PAGE and immunoblotting

For each sample, a volume corresponding to a normalized protein amount was supplemented with 10X SDS sample buffer (500 mM Tris pH 6.8, 25% SDS and 2% bromophenol blue in 70% glycerol-30% dH2O), NuPAGE Sample Reducing Agent (10X) (Thermo Fisher, NP0004) and distilled water to an equal final volume. Samples were denatured and reduced at 90°C for 5 minutes and run on Novex 4-20% Tris-Glycine and Novex 12% Bis-Tris mini gels *and 4-20% BioRad midi gels*. Activity-based probe fluorescence was measured either in gel or after transfer on the membranes on an Azure Sapphire biomolecular imager. For silver staining the gels were processed as described in the kit’s technical bulletin (Thermo Fisher, 24612). For immunoblot analyses, the gels were wet-transferred onto Immobilon-FL PVDF membranes (Sigma-Aldrich, 05317-10EA) or semi-dry transferred onto 0.2 μm nitrocellulose membranes (BioRad, 1704159). Equal loading and transfer were then assessed by Revert 700 total protein stain (LI-COR, 926-11011) or Ponceau stain (CST, 59803). The membranes were destained, blocked for 1h at room temperature in Intercept (TBS or PBS) blocking buffer (LI-COR, 927-60001, *927-70001*) and probed with primary antibodies overnight at 4°C. The next day the membranes were developed with fluorescently labeled secondary antibodies on a LI-COR Odyssey Classic system. The following primary antibodies and corresponding dilution factors were used: anti-PSMA3 (Enzo, BML-PW8110-0100 – ms, 1:1000), anti-PSMB8 (Enzo, BML-PW8845-0100 – ms, 1:1000), anti-PSMB5 (CST, 12919 – rb, 1:1000), anti-PSME1 (Abcam, ab155091 – rb, 1:1000), anti-Histone H3 (Abcam, ab1791 – rb, 1:1000), anti-TH (SYSY, 213104 – gp, 1:1000), anti-PSD95 (Abcam, ab2723 – ms, 1:1000, used for Proteasome validation), anti-PSD95 (Thermo Fisher, MA1-046, ms, 1:1000, used for synaptosome prep analysis), anti-Mbp (Abcam, Ab62631– ms, 1:1000), anti-GFAP (Abcam, Ab7260 - rb, 1:1000), anti-Syn (SYSY, 106002, rb, 1:1000), anti-SYPH (Sigma, S5768, ms, 1:5000), anti-Gad1/2 (Enzo, ADI-MSA-225-E, ms, 1:1000), anti-Syt12 (SYSY, 299 003, rb, 1:1000), anti-Proton ATPase (SYSY, 109 003, rb, 1:1000), anti-Complexin-1/2 (SYSY, 122 102, rb, 1:1000), anti-Stx1a (SYSY, 110 111, ms, 1:1000), anti-Stx1b (SYSY, 110 403, rb 1:1000), anti-Syt2 (SYSY, 105 223, rb, 1:1000), anti-Rab3c (SYSY, 107 203, rb, 1:1000), anti-Vamp1 (SYSY, 104 002, rb, 1:1000), anti-Vamp2 (SYSY, 104 202, rb, 1:1000), anti-TH (SYSY, 213 104, gp, 1:1000), anti-Oxr1 (Abcam, ab251774, rb 1:1000), anti-Mapk3 (CST, 9101, rb, 1:1000) and anti-Atp6v1g1 (ProteinTech, 16143-1-AP, rb, 1:1000). Normality of the data was assessed using a Shapiro-Wilk test. The homogenate and the synaptosome fraction F2/3 were compared using a paired one tailed t-test or the non parametric Wilcoxon signed rank test. P-values <0.05 were considered significant

#### Electron microscopy

Electron microscopy (EM) analysis of unsorted synaptosomes was conducted as described in Sebring et al.^74^ with chemicals from Sigma Aldrich and Plano GmbH (Pioloform). F2/3 fractions were fixed with EM grade glutaraldehyde (final concentration 2.5%) for 30 minutes on ice and, subsequently, diluted in PBS. During the fixation period, 5% agarose pucks were prepared by aspirating with a P10 pipette tip. Next, the pipette tip was lodged onto a stereological pipette tip allowing for a larger volume to be spun down onto the agarose puck. The fixed synaptosomes were spun onto the agarose puck using Labofuge 400R swinging bucket centrifuge at ∼4’000g for 1 hour at 4°C. Then, the liquid was aspirated carefully and the agarose puck was pushed out of the pipette tip and encapsulated in an agarose droplet. This droplet was stored at 4°C in PBS until further processing.

Sorted synaptosomes were processed as described above with minor alterations. First, ∼10 million synaptosomes were sorted into bovine albumin serum in PBS (final concentration 2%). The pipette tip containing the agarose puck was attached to a 15ml Falcon tube to accommodate the higher volume. Synaptosomes were spun down onto the agarose puck at 4°C and 4000g for 3 hours after which glutaraldehyde was added to a final concentration of 2.5%. The synaptosomes were fixed during centrifugation at 4°C at ∼4000g for 30 minutes. Next, samples were washed in 0,1 M Cacodylate buffer (CD) at room temperature and samples were further stained in 1% OsO4 (in 0.1M CD) for 30 minutes. Samples were washed in 0.1M CD and water, and subsequently stained with 1% uranyl acetate (UA) (in water) in the dark for 10 minutes. Samples were washed with water and dehydrated by incubation in a series of ethanol buffers ranging from 30% to 100% ethanol.

For epoxy resin embedding, the samples were washed in dehydrated Propylenoxid and incubated in a 1:1 mixture of EPON:dehydrated Propylenoxid for 30 minutes at RT. Then, samples were left in EPON overnight at RT. The following day, excess agarose was trimmed and samples were placed in a silicone mold and EPON was polymerized for at least 48 hours at 60°C. 70 nm sections were generated using a Leica Leica Reichert Ultracut S Ultramicrotome with a DiATOME ultra 45° diamond knife and mounted onto a Pioloform film on copper grids. EM imaging was performed with a Zeiss LEO 912 AB Omega and a Sharp Eye TRS (2x2k) CCD camera. ImageSP was used to control the CCD camera and to make tilescans with 20% overlap at a magnification of 31’500X at 120 kEV for characterisation of F2/3 unsorted synaptosomes. FASS synaptosomes were imaged at 4100X magnification at 120 kEV. Tiles were stitched for visualization using TrakEM2 in ImageJ.^75^ For image analysis, ∼30 synaptosomes from each sample were randomly selected, blinded (using ImageJ plugin Blind Analysis Tools), converted to 8-bit and the image contrast was normalized to 0.35 saturation. The blinded images were manually annotated for size, mitochondrial area and the presence of an attached postsynaptic density, while the number of SVs was determined with the automatic detection pipeline.^76^ Unblinding and subsequent analysis was performed in R. To estimate the true diameter, overcoming the random cross sectional nature of 2d TEM, the assumption of perfectly spherical synaptosomes was made. This simplification allowed for sampling random cross sections from a sphere with a known radius (R) by solving for Y in X^²^ + Y^²^ = R² given randomly drawn X values from the range 0-R. Then 2Y was used as the diameter of the randomly sampled cross section of the sphere. The above described was performed 30 times for four simulated samples.

Data was tested for extreme outliers, defined as outside the first quartile minus 3 x the interquartile range or third quartile plus 3 x the interquartile range, and for normality using a Shapiro-Wilk test. An ANOVA was used to compare synaptosome ultrastructural features across brain regions. To compare the cross sectional area of synaptosomes with a postsynaptic density (PSD) and without a PSD, a paired two-tailed T-test was used. P-values <0.05 were considered significant.

#### Fluorescence-activated synaptosome sorting

Fluorescence-activated synaptosome sorting (FASS) was performed as previously described.^19^^,^^21^^,^^22^ F2/3 fractions from the synaptosome preparation were diluted 1:50 in the GM buffer and 1.5μg/ml membrane dye (FM4-64, Thermo Fisher) was added. Synaptosomes were analyzed and sorted on a FACSAria Fusion (BD Biosciences) running FACSDiva, equipped with a 70μm Nozzle and the following settings: 488nm laser (for FM4-64), 561nm laser (for TdTomato), sort precision (0-16-0), FSC (317 V), SSC (488/10 nm, 370V), PE “TdTomato” (586/15 nm, 470V), PerCP “FM4-64” (695/40 nm, 470), thresholds (FSC = 200, FM4-64 = 700). Samples were analyzed and sorted at approx. 20’000 events/s and a flow rate of < 3. Doublet particles were excluded based on SSC-H and SSC-W. We constructed a gate (P3) to obtain the synaptosome population that selects particles that are double-positive for TdTomato and FM4-64 by thresholding against synaptosomes from wt mice (Figure 1B). For control synaptosomes all non-doublet particles above the FM4-64 threshold were sorted (P2). For each sorted sample (P3) as well as the matching control sample (P2) we sorted 20 Mio particles (Experiment described in Figure 1), 10 Mio particles (Experiment described in Figure 2A) or 2 Mio particles (Experiments described in Figures 6 and S9).

#### Synaptosome immunofluorescence

Sorted synaptosomes were centrifuged onto gelatin coated coverslips (Electron Microscopy Sciences) for 20 min at 4’000g and 4 °C in a Labofuge 400R and fixed for 15min in 4% PFA, 4% sucrose in PBS. Synaptosomes were then immunolabeled according to the manufacturer's instructions (Alexa Fluor 488 Tyramid SuperBoost Kit, Invitrogen, B40941) using the following antibodies: anti-PSD95 (Thermo Fisher, MA1-046, ms, 1:1000) and anti-Gephyrin (SYSY, 147011, ms, 1:1000), and subsequently mounted and imaged as described above for brain sections. Synaptosome co-localization analysis was performed in ImageJ using the SynaptosomesMacro published by Paget-Blanc et al.^19^

#### Synaptosome processing for MS

Sorted synaptosomes and control particles were filtered onto Whatman glass microfiber filters (GF/F, Cytiva) and stored at -80 °C until further processing. For filtration, a miniaturized custom-built apparatus was constructed. Synaptosomes were sorted directly into a 15ml tube (Protein LoBind, Eppendorf) that was connected through tubing (from an infusion set) to a luer connector unit with a 4mm diameter filter fixed between the male and female luer connector units. The tubing and filtration unit was cooled on ice and negative pressure was applied for filtration. Early experiments revealed the necessity of washing the filters with PBS to remove residual sheath fluid (BD FACSFlow), and as a result many of earlier samples were not analyzed. Samples from the experiment across brain regions were allocated to blocks and batch-processed. Synaptosome samples with 10 Mio or 20 Mio particles were lysed in 20μl lysis buffer (100 mM Tris, 1% sodium deoxycholate, 10 mM TCEP, 15 mM 2-chloroacetamide) by repeated sonication using a VialTweeter (Hielscher Ultrasonics), and heated to 95 °C for 5 min. Proteins were digested with 0.1μg LysC and 0.1μg trypsin (Promega) overnight at 37 °C. Samples were then acidified with 10% formic acid to approximately pH 3, and centrifuged for 10 min at 16’000 g. The supernatant was desalted using ZipTip pipette tips (Merck) and dried in a vacuum centrifuge. Synaptosome samples with 2 Mio particles were solubilized in 20ul TEAB buffer (50mM Triethylammonium bicarbonate, 1mM CaCl_2_, 0.05μg trypsin LysC and 0.05μg trypsin) and digested overnight at 37 °C. 30μl acetonitrile was added and the samples were centrifuged 10 min at 16’000 g. The supernatant was filtered through ZipTip pipette tips by centrifugation for 1 min at 2’000g and then 50μl of 50% acetonitrile in MS-grade water was added to the filter and both steps were repeated. Samples were dried in a vacuum centrifuge and stored at -20 °C until Liquid chromatography–tandem mass spectrometry (LC-MS/MS) analysis.

#### LC-MS/MS analysis

All samples were analyzed using label-free quantification in DIA-mode with targeted feature extraction using sample-specific spectral libraries built by DDA runs of in-house generated synaptosome samples. This approach was chosen to assure high sensitivity and reproducible coverage across a high number of relatively heterogeneous samples.^29^^,^^30^ The peptide samples were reconstituted in 5% acetonitrile (ACN) and 0.1% formic acid (FA) supplemented with an iRT peptide standard (Biognosys). Peptide mixtures were analyzed using an UltiMate 3000 nano-LC coupled to a Fusion Lumos mass spectrometer (Thermo Fisher Scientific). Briefly, samples were loaded onto a PepMap 100 C18 trap column (75 μm id × 2 cm length, 3 μm particle size; Thermo Fisher Scientific) at 6 μL/min for 6 min with 2% acetonitrile (v/v) and 0.05% trifluoroacetic acid (v/v) followed by separation on an C18 analytical column (75 μm id × 50 cm length, 1.7 μm particle size; CoAnn Technologies) maintained at 55°C. Peptides were separated by a non-linear 120 min gradient^30^ using mobile phase A (100% H2O, 0.1% formic acid) and B (80% acetonitrile, 0.1% formic acid). The Fusion Lumos mass spectrometer was operated in DDA mode for spectral library generation and DIA mode for all samples used in this study, using acquisition methods that are described in Muntel et al.^30^ In brief, the 40W DIA-method had the following settings: Full scan; orbitrap resolution = 120k, AGC target = 125%, mass range = 350-1650 m/z and maximum injection time = 100 ms. DIA scan; activation type: HCD, HCD collision energy = 27%, orbitrap resolution = 30k, AGC target = 2000%, maximum injection time = dynamic. The mass spectrometry proteomics data have been deposited to the ProteomeXchange Consortium via the PRIDE^68^ partner repository with the dataset identifier PXD039946.

#### Data analysis of DIA LC-MS/MS

LC-MS/MS samples were analyzed with Spectronaut version 16 (Biognosys) as previously described.^77^^,^^78^ Briefly, a spectral library was generated from collected in-house generated DDA FASS synaptosome files. The collected DDA spectra were searched against the UniProtKB/Swiss-Prot database for mus musculus (retrieved in May 2021) using the Sequest HT^79^ search engine within Thermo Proteome Discoverer 2.4 (Thermo Fisher Scientific). For samples that did not undergo alkylation by 2-chloroacetamide (samples with 2 Mio synaptosome input, see above), carbamidomethylation at cysteines was set as variable instead of fixed modification. The identified proteins were assessed using Percolator and filtered using the high peptide confidence setting in Proteome Discoverer 2.4. Analysis results were then imported to Spectronaut 16 for the generation of spectral libraries. Targeted data extraction of DIA-MS acquisitions was performed in Spectronaut 16 with default settings. The proteotypicity filter “only protein group specific” was applied. Extracted features were exported from Spectronaut for statistical analysis with MSstats 4^59^ using default settings. Briefly, for each protein, features were log-transformed and fitted to a mixed effect linear regression model for each sample. For significance testing, we required a minimum of 6 features (combination of precursor and fragment ion) and 12 measurements per protein per condition. The model estimated fold change and statistical significance for all compared conditions. For the P2 control conditions, all P2 samples were grouped according to the brain region of origin, independent of the mouse line. The Benjamini–Hochberg method was used to account for multiple testing and p-value adjustment was performed on all proteins that met the fold-change cutoff. Significantly different proteins were determined using a threshold fold-change >1.1 and adjusted p-value < 0.05. For analyses comparing conditions originating from the same brain region, the significance testing result and sample quantification (protein abundance) files from MSstats 4 were used. For analyses comparing multiple conditions across brain regions, we calculated the normalized log_2_(fold-change) of the matched P3/P2 pairs for every sample and protein, which was then used for further analysis.

#### Data analysis of synaptic proteomes

The synaptic proteomes for Camk2a+, Gad2+ and Syn+ proteomes were determined by quantitative enrichment against the P2 (unsorted) control samples and the other synapse types originating from the same brain region. Specifically, for a given brain region, the Camk2a+ the proteomes, for example, were defined as proteins that satisfy at least one of following criteria: (I) they met the fold-change and p-value cutoff comparing P3 (sorted) with P2 (unsorted) samples of the same brain region and were not significantly different between Camk2a+ and Gad2+ or (II) proteins that met the fold-change and p-value cutoff comparing Camk2a+ over Gad+ and had a positive fold-change comparing P3 (sorted) with P2 (unsorted) samples of the same brain region or (III) proteins that met the fold-change and p-value cutoff comparing Camk2a+ over Syn1+ and had a positive fold-change comparing P3 (sorted) with P2 (unsorted) samples of the same brain region. For Gad2+ and Syn1+ the proteomes were determined according to the above description. The Dat+,cortical interneuron and hippocampal subfield proteomes were defined by quantitative enrichment against the P2 control samples using the above mentioned cutoffs. To compare Gad2+ enriched synaptic proteomes between hippocampal subfields, we defined interaction terms in the linear model used in MSstats analogous to: Gad2+SubfieldA vs. Gad2+SubfieldB = (“Subfield A Gad2+” - ”Subfield A control”) - (“Subfield B Gad2+” - “Subfield B control). All further analysis was conducted using quantitative values obtained from MSstats 4 in the R environment (R studio 2023.03 running R 4.2). Variance partitioning was analyzed using the VariancePartition R package.^80^ The chord diagram was generated using the circlize R package.^81^ Protein to gene name conversion was done using either org.Mm.eg.db^82^ or the UniProt API. SynGO analyses were performed using the SynGO web page with default settings.^5^ Euler diagrams were constructed using the eulerr R package.^83^ STRING^31^ networks were generated in Cytoscape 3.9.1 using the Cytoscape StringApp^84^ and analyzed using CentiScaPe.^85^ Protein complexes were annotated using the CORUM database.^34^ Weighted gene correlation network analysis was done using the WGCNA R package^33^ using soft power 6, a signed hybrid network type and the bicor correlation function. Heatmaps were visualized using ComplexHeatmap.^86^ The adjacency matrix was visualized as a protein-protein correlation network in Cytoscape with a frequency cutoff of 0.3. Gene set enrichment analysis (GSEA)^87^ for Gene Ontology terms^88^ and KEGG pathways^89^ was performed with clusterprofiler.^90^ ggplot2 was used for visualization.^91^ Cartoons were generated using Adobe Illustrator 2023 or Biorender.com. FACS plots were generated with FlowJo 10.

#### Co-immunoprecipitation

The PSME1 co-immunoprecipitation was performed using a commercial immunoprecipitation kit (Abcam, ab206996) following the manufacturer's instructions. Briefly, DIV 28 rat cortical neurons were washed three times in cold PBS and lysed in RIPA buffer supplemented with protease inhibitors (Thermo Fisher, 78430). The lysates were clarified by centrifugation at 104 x g for 10 min at 4C and the protein concentration measured using the Precision Red protein assay (Cytoskeleton Inc., ADV02). For each co-immunoprecipitation reaction 500 μg of protein were used in a total volume of 500 μl. The antibodies used and corresponding dilution factors were: rabbit anti-PSME1 (Abcam, ab155091, 1:100), rabbit anti-PSME1 (Cell Signalling, 2408, 1:100) and rabbit isotype control (Cell Signalling, 3900, 1:200). The reactions were incubated on a wheel overnight at 4C and the next day capture was performed using 30 μl of agarose resin for 2h at room temperature. After five washes in wash buffer and two additional washes in PBS the proteins were eluted by boiling in 2X SDS sample buffer.

### Quantification and statistical analysis

Different appropriate statistical methods were used for different experiments and are indicated in the relevant sections of the STAR Methods section and the figure legends. Statistical analysis of mass spectrometry data was performed with MSstats 4^92^ in the R environment (R studio 2023.03 running R 4.2)

### Additional resources

We created an interactive web-tool that allows one to query the abundance and localization of individual proteins in individual synapse types and also download our data (The Synaptic Diversity Hub, https://syndive.org/).

## Data Availability

• The mass spectrometry proteomics data have been deposited to the ProteomeXchange Consortium via the PRIDE^68^ partner repository with the dataset identifier PXD039946. • This paper does not report original code. • Any additional information required to reanalyze the data reported in this paper is available from the lead contact upon request.

## Supplemental information

Table S1. Protein quantification of cortical Camk2a+ and Gad2+ synaptic proteomes, related to Figure 1 (A) Significance testing result file for the comparison Camk2a vs. unsorted controls (P2). (B) Significance testing result file for the comparison Gad2 vs. unsorted controls (P2). (C) Significance testing result file for the comparison Camk2a vs. Gad2.

Table S2. Protein quantification for synapse-enriched proteins including SynGO annotation, related to Figure 2 (A) List of all synapse-enriched proteins including quantitative information. (B) Overlap between synapse-enriched proteins and the SynGO database. (C) Enrichments for top-level SynGO terms for the 15 synapse subtypes.

Table S3. WGCNA adjacency matrix and correlations, related to Figure 4 (A) Adjacency matrix from WGCNA. (B) List of protein correlations with vGAT immunofluorescence and protein module associations. (C) List of protein correlations with vGlut1 immunofluorescence and protein module associations. (D) List of negatively correlated proteins that are members of a shared protein family.

Table S4. Protein quantification for hippocampal subfield analysis, related to Figure 6 (A) List of Gad2+ hippocampal subfield-enriched synaptic proteins. (B) Significance testing result file for the comparison of unsorted CA3 vs. unsorted CA1. (C) Significance testing result file for the comparison of unsorted DG vs. unsorted CA1. (D) Significance testing result file for the comparison of unsorted DG vs. unsorted CA3. (E) Significance testing result file for the comparison of Gad2+ CA1 vs. Gad2+ CA3. (F) Significance testing result file for the comparison of Gad2+ CA1 vs. Gad2+ DG. (G) Significance testing result file for the comparison of Gad2+ CA3 vs. Gad2+ DG.

Table S5. Protein quantification for cortical inhibitory subtypes, related to Figure 6 (A) List of cortical inhibitory subtype-enriched proteins including quantitative information. (B) Significance testing result file for the comparison of SST vs. VIP. (C) Significance testing result file for the comparison of SST vs. PV. (D) Significance testing result file for the comparison of PV vs. VIP.
